# Comparative Analysis of Japanese Three-Spined Stickleback Clades Reveals the Pacific Ocean Lineage Has Adapted to Freshwater Environments while the Japan Sea Has Not

**DOI:** 10.1371/journal.pone.0112404

**Published:** 2014-12-02

**Authors:** Mark Ravinet, Naoko Takeuchi, Manabu Kume, Seiichi Mori, Jun Kitano

**Affiliations:** 1 Ecological Genetics Laboratory, National Institute of Genetics, Mishima, Japan; 2 Biological Laboratory, Gifu-keizai University, Ogaki, Japan; Glasgow Caledonian University, United Kingdom

## Abstract

Divergent selection and adaptive divergence can increase phenotypic diversification amongst populations and lineages. Yet adaptive divergence between different environments, habitats or niches does not occur in all lineages. For example, the colonization of freshwater environments by ancestral marine species has triggered adaptive radiation and phenotypic diversification in some taxa but not in others. Studying closely related lineages differing in their ability to diversify is an excellent means of understanding the factors promoting and constraining adaptive evolution. A well-known example of the evolution of increased phenotypic diversification following freshwater colonization is the three-spined stickleback. Two closely related stickleback lineages, the Pacific Ocean and the Japan Sea occur in Japan. However, Japanese freshwater stickleback populations are derived from the Pacific Ocean lineage only, suggesting the Japan Sea lineage is unable to colonize freshwater. Using stable isotope data and trophic morphology, we first show higher rates of phenotypic and ecological diversification between marine and freshwater populations within the Pacific Ocean lineage, confirming adaptive divergence has occurred between the two lineages and within the Pacific Ocean lineage but not in the Japan Sea lineage. We further identified consistent divergence in diet and foraging behaviour between marine forms from each lineage, confirming Pacific Ocean marine sticklebacks, from which all Japanese freshwater populations are derived, are better adapted to freshwater environments than Japan Sea sticklebacks. We suggest adaptive divergence between ancestral marine populations may have played a role in constraining phenotypic diversification and adaptive evolution in Japanese sticklebacks.

## Introduction

Colonisation of new environments can lead to adaptive divergence, the evolution of reproductive isolation and progression towards speciation within evolutionary lineages [1]–[3]. Divergent selection between populations in different habitats that leads to adaptive divergence occurs when ecological opportunity arises – *i.e.* previously unused niche space becomes available. Yet adaptive divergence and diversification is not ubiquitous; for example Darwin's finches have evolved remarkable phenotypic diversity in foraging behaviour and morphology, while Galápagos' mockingbirds have not [4], [5]. Similarly, the extent of adaptive divergence is variable in three-spined stickleback lineages; with strong reproductive isolation and phenotypic divergence in some regions but not others [6], [7].

Many potential factors may constrain phenotypic diversification and adaptive divergence [5]. Intrinsic factors such as differences in rate of dispersion and adaption to novel niche space can shape both colonisation history and rates of evolutionary diversification. The genetic basis of adaptation is also important; adaptation from standing genetic variation is more rapid than that from *de novo* mutations [8]. Extrinsic factors such as competition with earlier colonizers may also prevent lineages from establishing [5], [9], [10]. Alternatively, constraint may simply arise due to historical contingency; *e.g.* the chance formation of novel island and lake environments and in turn, chance colonisation [4], [5], [9]. Most likely, differences in the extent of adaptive divergence arise because of a combination of these factors and considering interactions between them is a more informative approach for understanding constraint [6], [11], [12]. For example, when resources in the new environment are similar to those in the ancestral habitat, probability of survival and establishment of a species upon colonization is greater [10], [13]. Comparative analyses of genetic and phenotypic variation between lineages undergoing different rates of adaptive divergence and phenotypic diversification may help us better understand these constraints.

The formation of freshwater lakes and rivers by glacial and interglacial cycles has repeatedly created novel unoccupied niche space during the late Quaternary [14]. Several marine taxa, such as crustaceans, molluscs, annelids, and teleosts have colonized these newly formed freshwater environments and undergone adaptive divergence, phenotypic diversification and in some cases parallel speciation [14], [15] The three-spined stickleback (*Gasterosteus aculeatus*) offer an excellent example with repeated colonisation of freshwater environments throughout the Pleistocene resulting in parallel phenotypic adaptation and genomic divergence [3], [16], [17]. Adaptive divergence between stickleback species pairs is widespread throughout the distribution of the species [18]; however, the nature and extent this divergence can vary across spatial scales and between evolutionary lineages [6], [7], [12], [19]. For example, stronger phenotypic differences between freshwater ecotypes in Canada relative to Europe has led some authors to suggest genomic constraints arising from different evolutionary histories may be influencing divergence [6], [7].

Despite freshwater colonization being characteristic of three-spined sticklebacks [3], [16], [17], the Japan Sea three-spined stickleback lineage contains no freshwater populations at all [20], [21]. Freshwater populations consist only of individuals from the Pacific Ocean lineage and demonstrate considerable phenotypic diversity in gill raker number, body size and armour traits, much like freshwater populations occurring elsewhere in the stickleback distribution [17], [22]. Japan Sea stickleback populations in contrast show little phenotypic diversity throughout their distribution and are characterised by a smaller body size and smaller lateral armour plates in comparison to similarly anadromous Pacific Ocean fish [20], [22]. In Japan and Eastern Asia, the Japan Sea and Pacific Ocean stickleback clades co-exist [20], [21], [23]. These two divergent lineages likely experienced a period of allopatric divergence during the geographical isolation of the Sea of Japan due to sea-level change 1.5–2 million years ago [22]. Phylogenetic analyses show that all Japanese freshwater populations analysed thus far are repeatedly derived from the Pacific Ocean lineage only [20], [21]. The apparent lack of freshwater colonisation within the Japan Sea lineage is intriguing as all other known evolutionary lineages of three-spined stickleback are able to adapt to freshwater [24]–[28]. This is also surprising because there are many freshwater lakes and rivers surrounding the Sea of Japan. Comparisons between the Japan Sea and Pacific Ocean lineages may therefore shed light on the factors that have caused differences in the ability to colonise empty niche space and evolve adaptive divergence in foraging traits in this stickleback system.

Both lineages have extant marine forms that breed in brackish waters and rivers, the Pacific Ocean and Japan Sea anadromous forms (PA and JA hereafter). These lineages are reproductively isolated from one another due to hybrid male sterility [22], [23]. Pacific Ocean freshwater populations (PF herein) also occur. Some spatial isolation during spawning also occurs as PA fish migrate to freshwater while JA remain at higher salinities [29], [30]. The two anadromous forms also differ in size, shape, diet and trophic traits such as gill raker number [22], [30]. However, detailed analyses of habitat, resource use and niche width have not been carried out. Furthermore, it is not known whether the magnitude of habitat divergence differs between sympatric PA and JA populations and allopatric populations of both forms (*i.e.* where only a single form occurs).

Focusing on the Japan Sea and Pacific Ocean stickleback lineages we first asked whether phenotypic and ecological diversification rates are higher in the Pacific Ocean lineage compared to the Japan Sea lineage. Using phylogenetic comparative methods we quantified diversification in trophic traits, including gill raker number and resource use in both anadromous and freshwater populations. We expected that divergence in trophic ecology would be consistent with foraging trait divergence between lineages. This first part of our study aimed to explicitly test the hypothesis that significant adaptive divergence in freshwater foraging traits – *i.e.* gill raker morphology and resource use - has occurred between marine and freshwater populations in the Pacific Ocean lineage. In contrast, we expected the Japan Sea lineage to lack phenotypic diversification because of a lack of adaptive divergence and our results strongly indicate this is the case.

Second, we asked how anadromous forms of the two lineages differ in trophic morphology, ecology and feeding behaviour, specifically focusing on sympatric (i.e. both forms co-occurring) and allopatric (i.e. only one form present) populations of JA and PA fish. Forms from both lineages migrate to coastal regions to spawn and previous research has suggested PA migrate further upstream than JA [30]. We hypothesised that ancestral and on-going adaptive divergence within the Pacific Ocean lineage has predisposed PA populations to exploit freshwater resources more frequently and efficiently than their JA counterparts. We also hypothesised that interactions between JA and PA forms in sympatry may further increase divergence in resource use. Our results confirm that PA populations do indeed exploit greater freshwater resources than JA sticklebacks in both allopatry and sympatry. However, we also found that JA populations exploited a greater proportion of freshwater resources when the PA was absent. We suggest that if adaptive divergence between marine and freshwater habitats occurred ancestrally in the Pacific Ocean lineage but not the Japan Sea lineage, this may have played a role in constraining phenotypic diversification.

## Methods

### Ethics statement

Animal use protocols were approved by the Institutional Animal Care and Use Committee of the National Institute of Genetics (23-15). Fish sampling in Hokkaido was conducted under a permit issued by Hokkaido Prefecture.

### Sample collection

**Figure 1:**
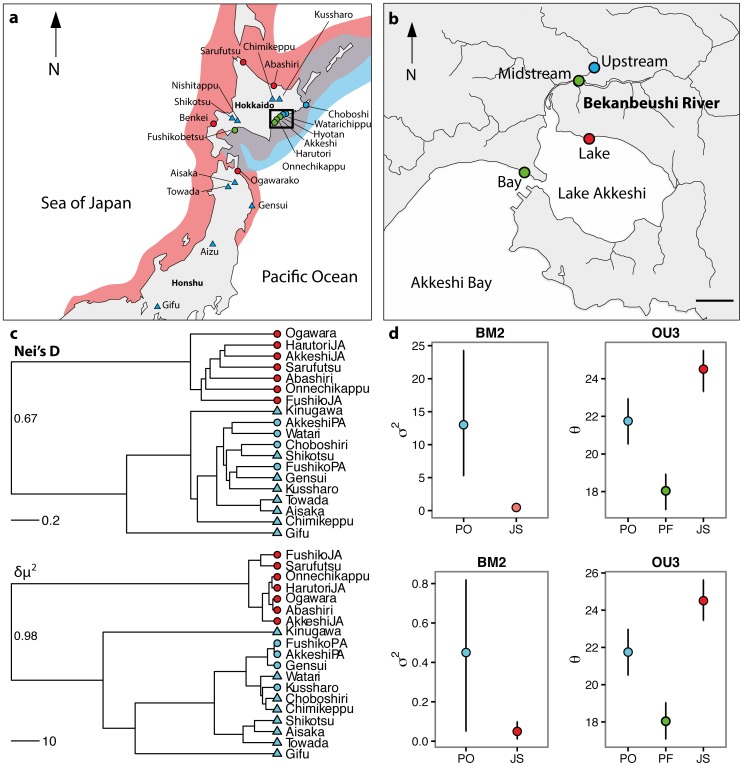
Maps showing a) sampling locations across Japanese archipelago, of Japan Sea (red) and Pacific Ocean (blue) stickleback distributions; red circles indicate allopatric Japan Sea anadromous, blue circles indicate allopatric Pacific Ocean anadromous, green circles denote sites with both forms present, blue triangles represent Pacific freshwater populations, black square indicates location of Akkeshi system in Eastern Hokkaido; b) sampling sites in the Akkeshi system; c) pruned phylogenetic trees for JS and PO populations where both stable isotope and gill raker data was available based on Nei's *D* and *δμ^2^*; bootstrap values based on 200 bootstrap iterations for JS and PO split are shown at tree root, see S2 for full bootstrap values on full phylogeny; d) mean rates of phenotypic evolution under BM2 (σ^2^) and OU3 (è) models (± bootstrapped 95% confidence intervals) for gill raker number. The upper panels are based on the Nei's D tree, while the lower panels are based on the *δμ^2^* tree.

Anadromous (PA, JA) and freshwater stickleback populations (PF) were sampled across Northern Japan using minnow traps and seine nets between June 2006 and May 2012 (Fig. 1a; Tables S1 and S22 in File S1). JA and PA fish were sampled from sites where both forms were present (sympatric) and also where only one form occurred (allopatric). In the Bekanbeushi (Akkeshi) system, Eastern Hokkaido (Fig. 1b), fish were collected clinally at three sites with increasing distance from the lake. Fish were immediately euthanized upon capture using MS-222, preserved in ethanol and classified as JA, PA or PF using morphologically identifying features [23].

Benthic macroinvertebrates were collected clinally from the lake in the Bekanbeushi and Shiomi river systems and marine invertebrates from Akkeshi Bay (Fig. 1b). Samples included putative prey items for stickleback as well as molluscs and bivalves to provide a representation of the baseline isotopic values of benthic and pelagic food webs [31].

### Trophic trait variation and stable isotope analysis

Dietary inference is an important means of determining habitat and resource use between divergent populations [30], [32], [33], [34]. While stomach content analysis provides a high-resolution indication of prey items [35] this method can only provide a temporal snapshot of resource use [36], [37]. In contrast, stable isotope analysis provides a long-term signal of diet and habitat use [38]–[40]. Using both stable isotope and stomach content analysis ensures characterisation of long-term diet and high resolution identification of prey items [35], [37].

The long-term resource use signal from stable isotope analysis is informative for anadromous species making large distance migrations across salinity gradients. As ectotherms, δ^13^C and δ^15^N values from fish muscle tissue typically reflect dietary assimilation during spring/summer growth as during winter nutrients are used to sustain basal metabolic processes [40]. For sticklebacks sampled in May-June shortly following the peak migration times for both forms [29], δ^13^C and δ^15^N values may indicate habitat divergence throughout life history. Three-spined sticklebacks from each of the sample sites were processed for stable isotope analysis (*n* = 314). Dorsal muscle was dissected from each fish, dried for 48 hours at 60°C, ground and weighed. Benthic macroinvertebrates (*n* = 66) were similarly processed. Samples were analysed for δ^13^C, δ^15^N, % C and % N on a Carlo Erba Elemental Analyser and a Thermo Finnigan Delta Plus XL mass spectrometer at the Duke Environmental Isotope Analysis laboratory (DEVIL) at Duke University, North Carolina, USA and at UC Santa Cruz Stable Isotope Laboratory, California, USA. Prior to analysis, fish muscle δ^13^C values were lipid-normalised [41].

Carbon and nitrogen isotopes can also be used to quantify the isotopic niche, a proxy for ecological niche [42], [43]. Niche quantification using Bayesian estimation of standard ellipses provides an accurate representation of niche width while quantifying sampling error [43]. We used SIBER (Stable Isotope Bayesian Ellipses in R) to estimate the corrected standard ellipse area (SEA_C_) and Bayesian estimated standard ellipse area (SEA_B_).

We additionally counted the number of gill rakers, a functionally important trait closely correlated with trophic ecology [32], [44]. Gill rakers were counted on the left first gill arch under a dissecting microscope (Tables S1 and S22 in File S1).

### Comparison of evolutionary rates

Two phylogenetic trees were estimated from microsatellite data in anadromous and freshwater populations from the Japan Sea and Pacific Ocean lineages using both Nei's *D* and *δμ^2^* [45], [46]. Briefly, fish (n = 249) were genotyped using 10 microsatellite markers (*Stn170*, *Stn233*, *Stn64*, *Stn159*, *Stn46*, *Stn90*, *Stn120*, *Stn278*, *Stn332* and *Stn384*) located on different three-spined stickleback linkage groups not linked to sex [23], [47]. While coalescent methods for estimation of population history from microsatellite markers are available [48], they do not integrate phylogenies across multiple markers and are not suitable for large numbers of populations. We used two metrics of genetic distance in order to account for the shortcomings of each metric; Nei's *D* performs best when divergence time is relatively recent whereas *δμ^2^* performs better when divergence is older [46], [49]. Pairwise matrices of genetic distances and UPGMA trees were estimated and bootstrapped 200 times using Populations [50]. Phylogenetic trees were then pruned using the R package *ape* so that only populations with ecological data remained (n = 19 for stable isotope data, n = 24 for gill rakers) [51].

To test for lineage specific rates of diversification for gill raker number and niche use (i.e. δ^13^C and δ^15^N) we first used the method developed by O'Meara et al. (2006). This allows phenotypic traits to evolve along a phylogeny under Brownian motion (BM) and estimates the likelihood of two models; a single rate only and separate rates (σ^2^) for the Japan Sea (JS) and Pacific Ocean (PO) lineages. Lineage was mapped onto each tree and nested Brownian motion models were fitted using the brownie.lite function in *phytools* [52], [53].

While widely applied, BM is a neutral model and may not be applicable when examining adaptive traits as it does not account for selection [54]. The Ornstein-Uhlenbeck (OU) model is an extension of BM including the parameters è and α; the optimum trait mean and the strength of selection against deviations from the optimum [54], [55]. Using Butler & King's (2004) method we tested OU models with single optimal trait value for both lineages (OU1), lineage specific optimal trait values (OU2) and lineage specific values with third optimal value for Pacific Ocean freshwater populations (OU3). If adaptive divergence has occurred between lineages, multiple optimum value models would be supported. As before, lineage was mapped onto the tree and nested OU models were fitted using the hansen function in the *ouch* R package [54].

Phylogenetic model choice is not straightforward as uninformative data may result in false positives using information criteria [56]. Through simulations under contrasting models, parametric bootstrapping produces likelihood ratios distributions which the observed data can be compared to, providing an estimate of power and a means to distinguish models [56]. Using the *pmc* R package [56] and custom functions, we performed parametric bootstrapping for the BM, OU and BM vs. OU tests based on 1000 simulated datasets. R scripts and datasets used to perform these analyses are available at the Dryad repository (doi:10.5061/dryad.s8f74).

Our final strategy was to perform both BM and OU tests on all trait and tree combinations choosing either a single or multiple parameter model based on the bootstrapped distributions. We then used bootstrapping to test whether it was possible to distinguish between the best-supported BM and OU models. Support for either a multiple rate BM model or a three optimum OU model would indicate a difference in diversification and adaptive divergence between the two stickleback lineages.

### Divergence in diet and feeding behaviour in a sympatric pair

Baseline-corrected δ^15^N values can provide an estimate of the trophic position of a consumer [31]. We collected molluscs (n = 15) and bivalves (n = 5) from the Bekanbeushi system to characterise the benthic and pelagic food webs and calculated trophic position (T_POS_) using Post's [31] method.

Isotope mixing models can estimate source proportions, providing a time-averaged indication of dietary preference [57]. To assess the contribution of marine vs. freshwater foraging environments to Japanese stickleback, we used mixing-models implemented in SIAR [58]. SIAR uses Bayesian inference to account for variation in sources and fractionation values allowing estimation of error and uncertainty. We estimated mean percentage contributions to each form at each site; contribution posterior probabilities were compared to test for differences.

Correlation between dietary preferences and ecomorphological traits is an proxy for detecting divergent natural selection between environments [34]. Stomach content analysis was performed on individuals sampled from Bekanbeushi (n = 284, see Table S20 in File S1 for site specific sample sizes). Fish captured in Akkeshi Bay had empty stomachs and were not included. Prey items were identified counted, weighed to the nearest 0.001 mg and then classified into seven categories; terrestrial insects, zooplankton, benthic macroinvertebrates, fish, fish eggs, plant material and other. Using frequency, weight and number we calculated the index of relative importance (%IRI_i_) [35].

We also conducted benthic foraging experiments to test whether freshwater foraging efficiency was greater in PA. Fish used were captured from Bekanbeushi system, returned to the laboratory and kept for one month. Experiments were conducted in a 63-litre clear glass tank (H: 35 cm, L: 60 cm, W: 30 cm) filled with 10% seawater. The tank was placed on the floor of a well-lit room with an ambient of temperature of 16°C. All sides apart from the front were covered to prevent startling the fish. Substrate consisted of fine sand and gravel, spread thinly to prevent benthic prey items from burying themselves beyond reach. Before the first trial on a given day, 60 live chironomid larvae were added to the test arena and spread at random across the substrate. Trials were then filmed using a SONY HVR HD 1000 placed at 1.5 m from the test tank. Trials were observed remotely and could be initiated, monitored and recorded without disturbing or startling the fish. A total of 42 trials were conducted over four days in June 2012 and the test arena was cleared, cleaned and refilled on each day.

Fish were fed a diet consisting of live *Artemia* and frozen chironomids for one week and were then starved for 24 hours before a trial to ensure feeding. Trials were conducted using the following protocol; a fish was chosen at random from a holding tank and then placed in the test arena containing food items. Once a fish made a vertical strike at a prey item, recording was started and a ten-minute foraging trial initiated. If no strike was made within 10 min after introduction, the trial was ended and the fish removed. Following trial completion, successful or not, fish were removed and their standard length recorded. Following each trial approximately five chironomid larvae were added to the test tank and the substrate raked to ensure prey visibility.

Measures of foraging efficiency were recorded from video footage. All vertical and horizontal strikes were counted; vertical strikes were defined as strikes made at a prey item resting on the substrate; horizontal strikes were defined as strikes made at prey suspended in the water column. The former captures the number of attempts to feed on new prey while the latter captures the number of strikes required to handle prey. Videos were reviewed again to calculate the number of chironomid larvae consumed per trial. The number of chironomids handled or abandoned was recorded and the difference equalled the number of chironomids consumed.

We calculated ratios of the number of vertical and horizontal strikes as well as the number of abandoned prey items to the number of chironomids handled in order to give an indication of handling efficiency. We additionally calculated a measure of foraging efficacy.
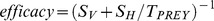
Where, *T_prey_* is total number of prey items consumed and *S_V_* and *S_H_* are vertical and horizontal strikes. This measure ranges from 0 to 1, with larger values indicating lower numbers of strikes per prey item consumed. We further calculated the number of vertical strikes per second to obtain a measure of foraging rate.

For SIA data, generalised linear mixed models (GLMMs) were used with species and site set as fixed or random factors depending on the test (specified in the *Results* section). Standard length and efficacy values were log_10_ transformed prior to analysis; all other foraging efficiency measures were square root transformed. Foraging efficiency measures were tested using GLMs with species as a factor and standard length as a covariate. All statistical analysis was conducted using R 2.15.1 [59].

## Results

### Higher phenotypic and ecological diversification rates in the Pacific Ocean clade than in the Japan Sea clade

Both Nei's *D* and *δμ^2^* trees clearly indicated two monophyletic groups with high bootstrap support, consistent with the Pacific Ocean and Japan Sea clades (Fig. 1c; Figure S2 in File S1). All freshwater populations occurred within the Pacific Ocean clade (Fig. 1c; Figure S2 in File S1), which is consistent with our previous findings that freshwater colonization occurs in the Pacific Ocean clade only [21].

Parametric bootstrapping indicated both phylogenies had high power to distinguish between single and separate rate Brownian motion models (i.e., different diversification rate between lineages; mean power 83%, Figure S3 & Tables S7 & S9 in File S1). BM2 models were highly supported for gill raker number and mean δ^13^C and δ^15^N values for both Nei's *D* and *δμ^2^* trees (mean power 99%, Table S7 in File S1). Therefore, diversification rates (σ^2^) for all three of these trophic traits were significantly higher for the Pacific Ocean lineage than the Japan Sea lineage (Fig. 1d, Table S9 in File S1), irrespective of phylogeny and sub-clade topology. Furthermore, this difference in diversification rate was not an artefact of sample size differences between lineages. Pacific Ocean σ^2^ values were higher than Japan Sea even when sample sizes for the two lineages were equal (Figure S11 in File S1). Nonetheless, power to detect these rate differences did increase with the number of Pacific Ocean populations included in the analysis (S13).

Power was also high in OU1 vs. OU2 comparisons (mean power 77%, Figure S4 & Table S7 in File S1), but less so for OU2 vs. OU3 (mean power 48%, Figure S5 & Table S7 in File S1). Multiple optimum models (OU2 & OU3) were highly supported for all trophic traits (Figures S5–S6 and Tables S7–S8 in File S1), suggesting that optimum trait values differed between lineages (Table S9 in File S1). For gill raker number, there was strong support for an OU3 model (Tables S7 & S8 in File S1) - i.e. lineage specific optimal trait values with an additional value for Pacific Ocean freshwater populations. Comparative results and power estimates were again consistent across topologies (Fig. 1C,D, Figures S11 & S12 in File S1) suggesting that low-topographic support within sub-clades (Figure S2 in File S1) did not influence our ability to detect higher diversification within the Pacific Ocean lineage. As with the BM models, sample size increased the variance between repeated analyses but did not alter the main findings that mean trait values differed between the lineages; Japan Sea gill raker length was consistently higher than Pacific freshwater populations when the number of Pacific Ocean populations included in the analysis was varied (Figure S12 in File S1).

Distinguishing between the best supported BM and OU models suggested that an OU2 model was preferable (mean power 97%, Figure S6 & Table S8 in File S1). Despite extremely high power (100%), it was not possible to distinguish between BM2 and OU3 models for gill raker number on either tree as the observed log likelihood ratio fell between test distributions (Figure S6 in File S1). This supports either increased trophic trait diversification or multiple adaptive optima within the Pacific Ocean lineage (Fig. 1d).

Mean gill raker number (è, lower – upper 95% CI) was lower in Pacific Ocean freshwater populations (18.04, 17.07–19.05) than in Pacific Ocean (21.75, 20-.50–22.99) and Japan Sea (24.51, 23.44–25.64) anadromous forms (Table S9 in File S1; also supported by GLMM with population as a random factor, P<0.0001). A strong positive correlation between mean gill raker number and δ^15^N (*r* = 0.70, *t* = 3.98, df = 17, *P*<0.001) was found, indicating a functional link to trophic ecology, although there was no δ^13^C correlation (*P* = 0.17).

### Ecological divergence in sympatry

**Figure 2:**
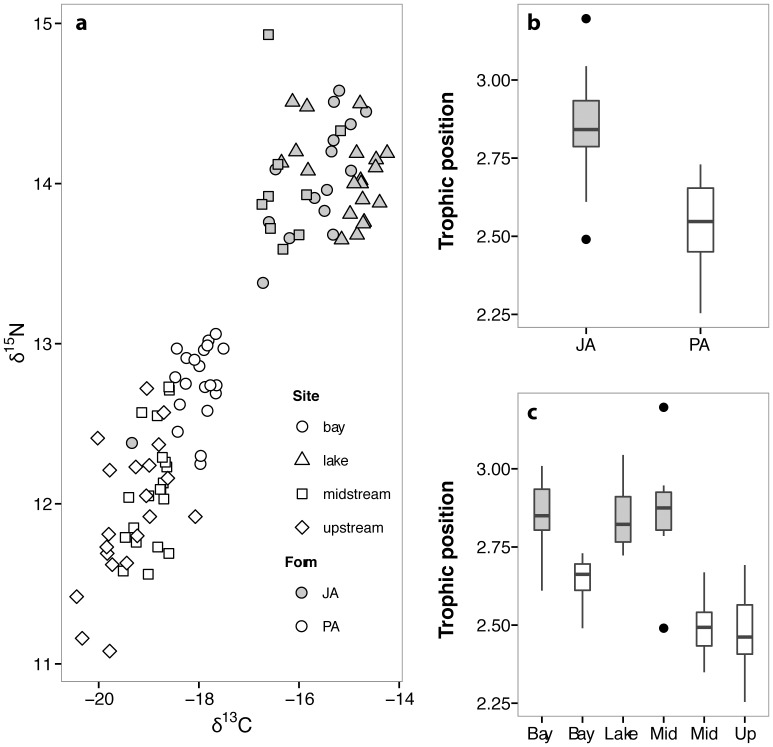
Stable isotope values for stickleback from the Bekanbeushi River system: a) δ^13^C and δ^15^N biplot; boxplots showing difference in trophic position between b) JA and PA forms and c) among forms captured at different sites within the catchment. NB: we are not able to explain why a single JA individual is present amongst the PA. Assignment was performed using species-specific microsatellites but we cannot rule out the possibility this sample was mislabelled.

To investigate ecological divergence between marine forms we first focused on a sympatric anadromous pair in the Akkeshi catchment. Stable isotope analysis on macroinvertebrate prey items indicated a clear transition from marine to freshwater environments in the Akkeshi catchment (Text S14 in File S1). Mean δ^13^C and δ^15^N values differed considerably between JA and PA forms (GLMMs, site as a random factor, δ^13^C: *F*_1, 100_ = 188.47, *R* ^2^ = 0.87, *P*<0.0001; δ^15^N: *F*_1, 100_ = 183.74, *R* ^2^ = 0.81, *P*<0.0001, Fig. 2a). Mean δ^13^C for PA was more depleted than that of JA, suggesting greater use of freshwater resources; in contrast JA showed general enrichment for δ^15^N over PA individuals (Table 1, Fig. 2a). Accordingly, mean trophic position was higher in JA stickleback (GLMMs with site as a random factor, *R* ^2^ = 0.81, *F*_1, 100_ = 119.93, *P*<0.0001, Fig. 2b).

**Table 1:** Mean stable isotope values for sympatric Japan Sea and Pacific Ocean stickleback from Akkeshi catchment; *n* indicates the number of individuals used for SIA.

Site	Form	*n*	δ^13^C	sd	δ^15^N	sd	T_POS_	sd
*Bay*	Japan Sea	16	−15.81	1.12	13.94	0.53	2.86	0.10
	Pacific Ocean	20	−17.99	0.29	12.76	0.23	2.64	0.07
*Lake*	Japan Sea	20	−15.05	0.63	14.04	0.26	2.84	0.10
*Mid*	Japan Sea	10	−16.50	0.92	13.82	0.72	2.86	0.18
	Pacific Ocean	19	−18.93	0.32	12.09	0.37	2.50	0.10
*Upstream*	Pacific Ocean	20	−19.39	0.61	11.94	0.44	2.48	0.11

Spatial variation in stable isotope values was also apparent within forms (Fig. 2a, c). JA stickleback captured at the midstream site were more δ^13^C depleted than conspecifics captured in the bay and lake (see Table 1, GLMM, collection year as random factor; *R* ^2^ = 0.31, *F*_2, 42_ = 9.01, *P*<0.0001). PA fish varied spatially for both δ^13^C (*R* ^2^ = 0.66, *F*_2, 55_ = 11.87, *P*<0.0001) and δ^15^N (*R* ^2^ = 0.52, *F*_2, 55_ = 4.87, *P* = 0.01). This was largely driven by δ^13^C variation between fish captured at the bay site and those in the river (*P*<0.05 in both cases). Focusing on the bay and midstream sites where both species co-occur, δ^13^C differences occurred between sites (GLM; *R* ^2^ = 0.76, *F*_3, 61_ = 67.34, *P* = 0.02) and forms (*P*<0.0001) but no significant interaction could be detected (*P* = 0.48), suggesting the difference between species did not vary between sites.

Bayesian source estimation revealed a greater contribution of marine benthic sources to JA fish (Table S15 in File S1, *P* = 0.002) while freshwater benthic prey was a more important prey resource for PA fish (*P* = 0.00, Tables S15 & S17, Figure S16 in File S1). Freshwater benthic contributions increased in both forms upstream (Table S1 & Text S14 in File S1). SEA_C_ values suggested no isotopic niche overlap for JA and PA fish (Tables S18 & S19 in File S1). SCA revealed some overlap (PSI = 0.20) and that both forms fed on planktonic and benthic prey items (Table S20 in File S1). However JA fish fed on larger proportions of zooplankton (%IRI = 73.35) than PA fish (%IRI = 0.14, *X* ^2^ = 72.94, df = 1, *P*<0.0001), consistent with a greater marine contribution to diet.

**Figure 3:**
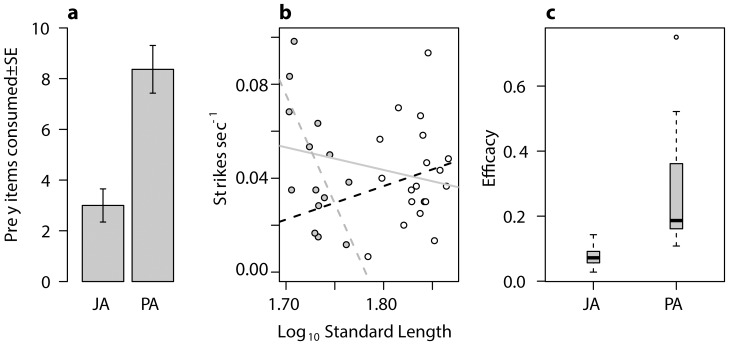
Measures of foraging efficiency for Japan Sea (JA) and Pacific Ocean (PA) anadromous stickleback; a) barplot showing mean values of chironomid larvae consumed; b) relationships between strike sec^−1^ and standard length, open and grey circles indicate PA and JA fish respectively, thick grey line represents common slope (R^2^ = 0.01, F_1, 32_ = 1.59 P = 0.21), dashed black and grey lines indicate JA (R^2^ = 0.31, F_1, 13_ = 7.46, P = 0.02) and PA (R^2^ = 0.00, F_1,17_ = 0.40, P = 0.53) relationships; c) boxplot showing differences in mean efficacy values.

To further confirm the divergence in trophic ecology between the sympatric PA and JA fish, we conducted a total of 42 benthic foraging trials (17 JA, 25 PA) on wild fish captured from the Bekanbeushi system (Fig. 1b) of which 35 were successful (6 PA failures, 1 JA). PA fish were larger than JA (standard length mm ± SD; 68.2±3.5 and 53.6±2.4, respectively; GLM *R* ^2^ = 0.86, *F*_1, 32_ = 205.9, *P*<0.0001) so size was included as a factor in the analysis to test for size specific effects. PA fish consumed a greater number of chironomids per trial than JA fish (GLM R^2^ = 0.39, *F*_1, 32_ = 22.02, *P*<0.0001, Fig. 3a, Table S21 in File S1) and also had greater efficacy values (*R* ^2^ = 0.48, *F*_1, 32_ = 30.95, *P*<0.0001, Fig. 3c). Although the number of strikes per second did not differ (*P* = 0.62), a significant interaction indicated a size effect (*R* ^2^ = 0.16, *F*_3, 30_ = 3.03, *P* = 0.009; Fig. 3b).

### Patterns of ecological divergence between two marine forms in Japan

**Figure 4:**
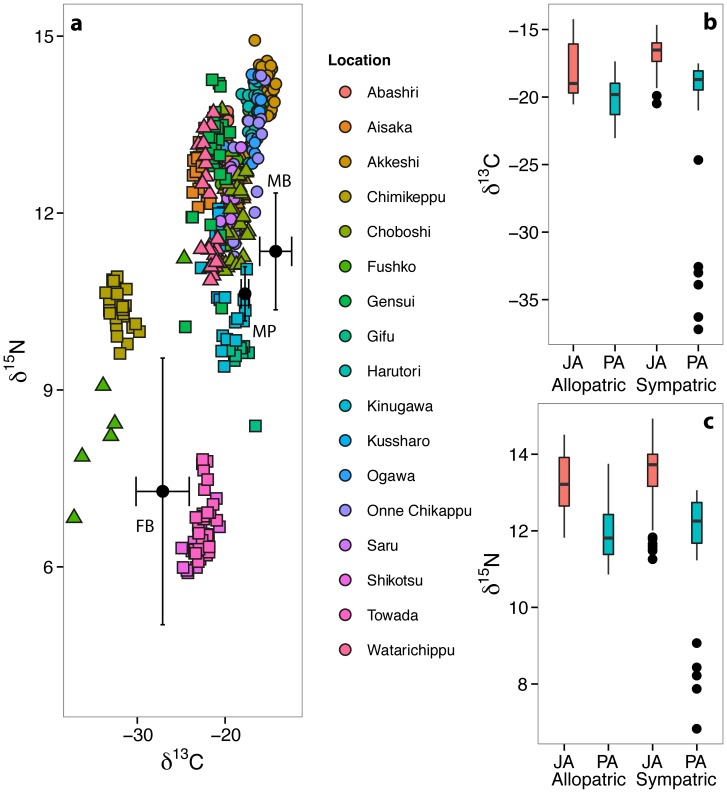
δ^13^C and δ^15^N isotope biplot (a) showing Japan Sea (circles), Pacific Ocean (triangles) and Pacific freshwater populations (squares; note, individuals from Fushikobetsu not shown to aid interpretation) with putative dietary source values (mean ‰ ± SD); boxplots (b and c) representing differences in δ^13^C and δ^15^N values between allopatric and sympatric JA and PA populations.

Comparing anadromous forms from both lineages across Japan, mean δ^13^C values for PA were lower than JA populations at both sympatric and allopatric sites, indicating greater freshwater foraging in the former (GLMMS with site as a random factor: *R* ^2^ = 0.86, *F*_1, 261_ = 77.46, *P*<0.0001, Table 2, Fig. 4a, b). JA fish also had higher mean δ^15^N values, suggesting feeding at a higher trophic level (*R* ^2^ = 0.62, *F*_1, 261_ = 79.28, *P*<0.0001, Table 2, Table S1 in File S1, Fig. 4a, c). These results suggest that trophic divergence and greater freshwater resource use in PA is consistent across the distribution range.

**Table 2:** Isotope niche metrics for allopatric and sympatric forms; SEA_C_ = corrected Standard Ellipse Area, SEA_B_ = Bayesian Standard Ellipse Area.

	δ^13^C (±SD)	δ^15^N (±SD)	SEA_C_	SEA_B_ (±SD)
JA	−17.31 (1.79)	13.36 (0.79)	3.66	3.70 (0.29)
PA	−20.18 (3.39)	11.94 (1.06)	8.46	8.81 (1.04)
PF	−23.28 (4.17)	9.85 (2.54)	6.14	6.35 (0.94)
Allopatric JA	−17.1 (2.09)	13.24 (0.75)	3.31	3.46 (0.40)
Sympatric JA	−16.85 (1.31)	13.48 (0.83)	2.16	2.26 (0.25)
Allopatric PA	−20.35 (4.86)	11.90 (1.38)	3.52	3.54 (0.44)
Sympatric PA	−20.34 (4.17)	9.86 (2.55)	6.12	6.74 (0.94)

We additionally tested whether allopatric (i.e. only a single lineage is present at a site) and sympatric (i.e. both lineages are present) JA and PA populations differed in trophic ecology. Distribution did account for δ^13^C variation (*P* = 0.02) and a significant species * distribution interaction (*P* = 0.05; GLM, *R* ^2^ = 0.24, *F*_3, 270_ = 29.42, *P*<0.0001) indicated that JA fish occurring in sympatry with PA fish had a more marine δ^13^C signal than allopatric JA populations; no difference occurred between allopatric and sympatric PA populations (Fig. 4b and c). Isotopic niche size was also larger in allopatric JA, allopatric PA and sympatric PA populations compared to sympatric JA (*P* = 0.99, 0.96 & 1 respectively, Table 2), suggesting a possible role of competition when JA and PA forms co-occur.

## Discussion

The results of our comparative phylogenetic analyses show that rates of phenotypic and ecological diversification are higher in the Pacific Ocean stickleback lineage compared to the closely related Japan Sea lineage. Since marine-freshwater adaptive divergence has only occurred in the former, our findings support the hypothesis that colonization of freshwater environments facilitates the evolution of increased diversity in stickleback foraging traits between marine-freshwater populations within lineages. Different mean optimal gill raker numbers between JA and PA populations indicate divergent ecological selection also occurs between these anadromous forms. To further investigate differences in optimal trophic trait values, we quantified divergence in resource use, foraging morphology and behaviour between the anadromous forms (PA and JA) in these two lineages. PA exploited more benthic freshwater resources, consistent with fewer gill rakers and improved prey handling on benthic macroinvertebrates compared to JA in both sympatry and allopatry. Stable isotope analysis also suggested allopatric JA populations exploit more freshwater resources when PA are not present, suggesting the possibility that competition may occur between the two anadromous forms. Together, our results confirm that only the Pacific Ocean lineage has undergone extensive diversification in foraging behaviour, ecology and morphology as a result of marine-freshwater adaptation and that substantial ecological divergence has occurred between Pacific Ocean and Japan Sea anadromous forms.

### Adaptive divergence has occurred in the Pacific Ocean lineage but not the Japan Sea lineage

Sticklebacks have been extensively studied as model organisms for studies of adaptive divergence and evolution [1], [16]. However most studies have focused on the ecological and genetic mechanisms underlying phenotypic diversification occurring after freshwater colonization [18]. In comparison we know little about the ecological and genetic mechanisms that might have constrained adaptive evolution in these species. Having failed to colonize freshwater environments and evolve increased phenotypic diversification, the Japan Sea lineage provides a unique opportunity to study why this is the case. Examining factors limiting adaptive evolution in this lineage may provide additional insight to factors promoting it in others.

Selection between different habitats, resources and niches is the major determinant of adaptive divergence between populations [1], [60]. Such adaptive evolution can result in different optimal traits amongst adaptive peaks, leading to an increase in the mean phenotypic diversity of a set of populations [1], [61]. Strong support for a BM2 model for trophic ecology and foraging morphology indicates that phenotypic diversity has evolved in the Pacific Ocean lineage but not the Japan Sea (Fig. 1D). Similarly, our comparative phylogenetic analysis also supported an OU3 model. This confirms adaptive divergence has occurred between both lineages and within the Pacific Ocean only with different adaptive optima for JA, PA and PF populations respectively (Fig. 1D). It should be noted that it was not possible for our analysis to effectively distinguish which of these two models provided a better fit for trophic morphology. However, either scenario - higher rates of evolutionary diversification or multiple adaptive optima - suggests foraging traits such as gill raker number have diversified within the Pacific Ocean lineage.

In addition to our comparative phylogenetic results, our other analyses and previous research provides a strong case for adaptive divergence between anadromous and freshwater resident populations of the Pacific Ocean lineage. Our stable isotope data showed clear structuring along a marine-freshwater axis, with obligate freshwater PF populations at one end and JA populations at the other (Fig. 4). While PA populations have a greater freshwater signal than JA populations, there is also dietary structuring between PA and PF populations. Furthermore, this marine-freshwater dietary structuring within the Pacific Ocean lineage is accompanied by phenotype-environment associations; ecologically functional traits such as reduction of gill raker number and body size in freshwater populations have been shown in both present and previous studies [22], [62]. Further work is necessary to directly confirm that these phenotype-environment associations are indicative of a direct functional link between foraging traits and improved fitness in marine and freshwater environments [1], [33], [63]. However, our stable isotope and behavioural data indicate improved freshwater foraging efficiency in the Pacific Ocean lineage. Given that there is considerable evidence that gill raker number and body size influences foraging efficiency in other stickleback populations [32], [44], [64], it seems likely this is also the case between PF and PA populations in Japan.

### Adaptive divergence between ancestral forms may have constrained colonisation of novel environments

Evolutionary diversification is closely linked to the colonization of new environments and establishment success often depends on the similarity of these environments to the source habitat as colonisers may already possess suitable adaptations [5], [13]. Divergence in habitat-specific adaptations between the ancestral anadromous populations of these lineages may therefore have played a role in constraining adaptive divergence in this case; i.e. adaptation to marine foraging in the Japan Sea lineage might result in an intrinsic constraint limiting freshwater resource use.

Our stable isotope and stomach content analyses demonstrated divergent resource use between anadromous forms from the two lineages. PA stickleback exploited a greater proportion of freshwater resources than JA across their distribution with mean isotopic values more similar to freshwater resident populations (PF). Spatial isolation of spawning sites following migration upstream between the PA and JA forms at sites where both forms co-occur is supported by our present stable isotope data and also by previous longitudinal demographic studies . Habitat isolation arising as a by-product of divergent natural selection occurs quite readily between stickleback species pairs [32], [65], [66] and the consistent pattern of divergence across the distribution of the Japan Sea and Pacific Ocean lineages suggests divergent selection might act on their anadromous forms.

Our comparative OU analysis supported a three optimum model for gill raker morphology – i.e. Japan Sea fish have a higher mean number of gill rakers than Pacific Ocean anadromous and freshwater populations. Support for an additional optimum in Pacific freshwater populations suggests freshwater colonization is characterised by a reduction in gill raker number and a shift towards a new adaptive peak [61]. Gill raker number is closely associated with determining foraging efficiency throughout the stickleback species complex [32], [33], [67]. Fish with numerous long gill rakers show greater efficiency when foraging for pelagic prey; whereas fewer, shorter gill rakers occur in benthic ecotypes [32]. This additional optimum value also indicates that adaptive diversification has occurred with the Pacific Ocean lineage but not the Japan Sea lineage.

Our behavioural experiment supports a functional link between gill raker morphology and foraging efficiency in the sympatric Bekanbeushi population. PA fish consumed more benthic prey and demonstrated improved prey handling. As variation in gill raker number was not included in our experimental design, we cannot conclude that lower efficiency with benthic prey in JA fish is as a result of morphological adaptation to feeding on pelagic prey. However gill raker number has been shown to correlate closely with foraging efficiency in stickleback and other fish species [44], [68]. Furthermore, our stable isotope data supports a strong correlation between foraging morphology and behaviour in Japanese sticklebacks; enriched mean δ^15^N values and higher trophic level in Japan Sea anadromous fish indicate increased pelagic diet. Correlations between morphology and behaviour are compelling but further work is necessary to test whether gill raker morphology directly increases foraging efficiency in the Japanese stickleback system.

Body size can also influence prey capture success in fishes and may be more important in determining benthic foraging success than trophic morphology [32], [44], [68]. A significant relationship between strikes per second and size suggests larger JA individuals exhibit improved prey handling. This was not the case for PA fish however as adult PA show very little body-size variation (J Kitano, personal observation) and our sample may have lacked the variation necessary to show such a relationship. Preliminary results from foraging experiments using smaller fish support this conclusion as both forms show poor benthic prey handling (Ravinet & Kitano, unpublished data). Smaller stickleback have a greater handling cost when feeding on larger food items such as benthic macroinvertebrates [69]. Since JA stickleback are smaller than PA [22], this may contribute to lower benthic prey foraging efficiency, suggesting size may act as a competitive advantage for PA when colonizing freshwater environments.

Although our present study has focused on ecological factors arising from differences in foraging and morphology, physiological constraint may have also played a role in preventing marine-freshwater adaptive divergence in the Japan Sea lineage. ‘Key innovations’ are adaptive traits which allow new niches to be exploited and can increase diversification, potentially leading to adaptive radiations when they arise [70], [71]. In contrast, the loss or failure to evolve key innovations would prevent a lineage from being exposed to divergent selection necessary for adaptive divergence and diversification. The Pacific Ocean lineage and all other known stickleback lineages have been able to colonise freshwater environments [21], [25], [27], [28]. Furthermore, adaptive divergence between stickleback species pairs occurs either between contrasting freshwater environments or along a marine-freshwater axis. Freshwater tolerance may therefore be a key innovation that has allowed increased diversification within the Pacific Ocean and other stickleback lineages. JA however exhibit lower freshwater tolerance with low survival rates in freshwater than PA [72], [73]. The loss or reduction of freshwater tolerance probably plays a major role in preventing JA fish from undergoing adaptive divergence.

### Possible extrinsic constraints of adaptive divergence: competition

Ecological interactions, such as competition for resource use, may also play an important role in shaping colonization of and adaptation to novel environments [9], [74]. More depleted δ^13^C values in allopatric JA populations suggest that the JA form is able to make greater use of freshwater resources when PA fish are absent. Competition for resources can also have a negative effect on population densities during colonization [74]. Where JA and PA fish occur in sympatry, numbers of JA fish are small and fluctuate yearly [30]. PA fish also display much better benthic prey handling than JA fish, suggesting that competition in sympatry may restrict JA habitat use, limiting freshwater adaptation and diversification.

Priority effects may also explain the difference in the ability of the two lineages to adapt to freshwater. Priority effects are species-specific interactions that influence establishment and fitness [75]–[77]. In short, if the Pacific Ocean lineage was able to reach freshwater environments before the Japan Sea lineage, this may have given it a fitness advantage. This seems unlikely as current evidence suggests the two lineages diverged 1.5–3 million years in the Japanese archipelago when the Sea of Japan was isolated from the Pacific Ocean during the Quaternary [20], [22], [23]. Furthermore, fossil evidence suggests sticklebacks have been present in East Asia and around Japan for ∼10 million years [78]. Additionally, freshwater environments occur on both the Pacific and Sea of Japan coasts of the Japanese Islands, indicating both lineages would have been able to access freshwater environments during and following divergence.

Competition may play some role in determining differences in resource use when the two lineages occur in sympatry, however it seems unlikely this is the sole explanation for the failure of the Japan Sea lineage to diversify by colonizing freshwater environments, Resource competition would only prevent colonisation where the two forms overlap (Fig. 1a) and there is no evidence of freshwater colonisation in regions where Japan Sea fish are present and Pacific Ocean absent, despite suitable freshwater habitats being available [79]. Nonetheless the nine-spined stickleback (*Pungitius spp.*) could also act as a competitor, having adapted to freshwater environments connected to the Sea of Japan [80], [81]. Nine-spined stickleback are also absent from the Pacific coast, except in Hokkaido potentially allowing greater ecological opportunity for the Pacific Ocean lineage although this has not been explicitly studied. Further work is necessary to test whether inter-specific competition occurs between Japanese stickleback lineages.

Internal and external constraints on adaptation and phenotypic diversification should not be considered in isolation as the interaction between both classes of constraint is often more informative [11]. Intrinsic constraints for example may play a role in mediating ecological interactions increasing the effect size of potential external constraints. Low freshwater tolerance in Japan Sea sticklebacks [72], [73] for example would explain the lack of diversification in freshwater environments even in areas where the two forms do not overlap. An interesting avenue for further work is to test whether intrinsic constraints such as freshwater tolerance can influence ecological interactions such as competition and foraging efficiency. A combination of multiple factors, including intrinsic constraints and interspecific competition probably constrain freshwater colonisation and morphological diversification in the Japan Sea lineage [70].

### Importance of comparative studies of adaptive divergence

Genomic constraints on phenotypic diversification remain uncertain, although the loss of allelic variants underlying traits may constrain adaptive divergence [6]. Mutation effect sizes influence the probability of adaptation as genes of small effect are less likely to shift populations closer to distant adaptive optima [61], [82]. Divergent selection between lineages leading to stronger adaptation to the marine environment by the Japan Sea lineage may have driven it further from a freshwater optimum. Since multiple genes of small effect play a role in stickleback freshwater adaptation [83] this may have led to genomic constraint on diversification. Selection from standing genetic variation is also important for stickleback adaptation [8], [84]. Loss of genetic variation in genomic regions underlying adaptive traits during a period of isolation may have lowered adaptive potential in the Japan Sea lineage. Further work combining biogeographical information and next-generation sequencing is now necessary to identify the roles of selection and drift in the loss of adaptive genomic variation in the Japan Sea stickleback lineage.

Understanding factors that facilitate or constrain adaptive divergence, phenotypic diversification and adaptive radiation is a fundamental question for evolutionary biologists. Both phylogenetic and experimental data have demonstrated that ecology of founders can influence the patterns of adaptive radiation [85], [86]. Although an increasing number of studies have examined the genomic basis for adaptive evolution [87], [88], few have focused on lineages that are unable to diversify or undergo divergence. Comparing closely related lineages differing in the magnitude of phenotypic divergence is fertile ground for developing an understanding of the genetic constraints on adaptive evolution. Different mechanisms might constrain divergence and diversification in different taxa, particularly when chance and historical contingency play a greater role than the deterministic influences of selection [89]. Further studies on the differences in phenotypic diversification across diverse taxa are required for a better understanding the constraints on adaptive divergence following the colonisation of novel environments.

## Conclusions

Ecological opportunity is thought to be key for phenotypic diversification [4], [5]. Adaptive traits allowing the exploitation of new environments, are fundamental for driving adaptive divergence, radiation and phenotypic diversification [70]. Indeed, when ancestral species make transitions into new environments similar to their current habitat, survival and diversification is more likely [13]. Failure to maintain or evolve adaptive traits may constrain the colonization and invasion of new environments, ultimately limiting the range and evolutionary diversification of a species. Further comparative and genomic studies of closely related lineages with differing evolutionary rates will provide a promising means of understanding the constraints on diversification and adaptive evolution.

## Supporting Information

File S1 **Supplementary text, tables and figures.**
(DOCX)
