# Supplementary material for: Comparative Analysis of Japanese Three-Spined Stickleback Clades Reveals the Pacific Ocean Lineage Has Adapted to Freshwater Environments while the Japan Sea Has Not
Source: PLoS One. 2014 Dec 2;9(12):e112404. doi: 10.1371/journal.pone.0112404 (PMC4251985; doi:10.1371/journal.pone.0112404)
Supplement: File S1 — Supplementary text, tables and figures. (DOCX) [file pone.0112404.s001.docx]

**File S1**

Table S1: Sample site location and mean trophic trait values. Stable isotope values (δ^13^C and δ^15^N), Bayesian isotopic niche-width metrics and gill raker number; SEA^­^_B_ = Bayesian Standard Ellipse Area, GRN = Gill raker number. *n* indicates the number of individuals used for SIA.

| Site | Form | Latitude | Longitude | δ^13^C (±SD) | δ^15^N (±SD) | SEA_B_ (±SD) | *n* | GRN(±SD) |
| --- | --- | --- | --- | --- | --- | --- | --- | --- |
| Abashiri | JA | 43.960 | 144.200 | -19.86 (0.15) | 13.06 (0.37) | 0.58 (0.13) | 20 | 24.95 (1.93) |
| Aisaka | PF | 40.592 | 141.221 | -22.99 (0.57) | 12.67 (0.42) | 1.12 (0.27) | 18 | 17.00 (1.73) |
| Bekanbeushi | JA | 43.055 | 144.836 | -15.63 (1.04) | 13.96 (0.49) | 1.49 (0.23) | 46 | 24.98 (1.30) |
|  | PA | - | - | -18.77 (0.73) | 12.27 (0.51) | 0.92 (0.12) | 59 | 21.46 (1.33) |
| Chimikeppu | PF | 43.629 | 143.885 | -31.93 (0.99) | 10.34 (0.34) | 1.33 (0.27) | 25 | 20.8 (1.40) |
| Choboshi | PA | 43.258 | 145.556 | -18.81 (0.92) | 11.99 (0.74) | 2.32 (0.50) | 22 | 22.5 (1.73) |
| Fushikobetsu | JA | 42.540 | 141.333 | -18.00 (1.58) | 13.18 (0.62) | 3.28 (1.33) | 7 | 23.00 (1.55) |
|  | PA | - | - | -26.05 (7.24) | 10.31 (1.94) | 14.18 (4.11) | 13 | 21.29 (1.44) |
| Gensui | PF | 39.365 | 141.897 | -21.21 (1.06) | 12.89 (1.04) | 3.36 (0.68) | 25 | 19.75 (1.41) |
| Gifu | PF | 35.536 | 136.663 | -18.28 (0.95) | 9.50 (0.42) | 1.87 (0.67) | 9 | 13.80 (1.79) |
| Harutori | JA | 42.969 | 144.396 | -16.75 (0.80) | 13.72 (0.51) | 1.28 (0.24) | 30 | 25.35 (1.76) |
| Kinugawa | PF | 37.511 | 139.866 | -19.28 (1.47) | 10.21 (0.50) | 2.89 (0.83) | 13 | 17.90 (1.32) |
| Kussharo | PF | 43.600 | 144.348 | -20.76 (0.32) | 11.45 (0.49) | 1.04 (0.29) | 13 | 19.05 (4.37) |
| Ogawara | JA | 40.838 | 141.367 | -16.77 (1.03) | 13.59 (0.54) | 2.14 (0.60) | 14 | 24.46 (1.76) |
| Onnechikappu | JA | 42.966 | 144.116 | -17.66 (1.46) | 12.63 (0.96) | 3.39 (0.80) | 19 | 24.55 (1.28) |
| Sarufutsu | JA | 43.960 | 144.200 | -19.25 (0.45) | 12.37 (0.36) | 0.83 (0.19) | 20 | 24.25 (1.71) |
| Shikotsu | PF | 42.775 | 141.400 | -23.00 (1.22) | 6.40 (0.34) | 1.55 (0.35) | 20 | 15.40 (1.98) |
| Towada | PF | 40.445 | 140.842 | -22.49 (0.63) | 6.79 (0.54) | 1.25 (0.23) | 30 | 19.18 (1.31) |
| Watarichippu | PA | 43.036 | 145.053 | -21.71 (0.66) | 11.98 (0.96) | 1.98 (0.41) | 24 | 22.10 (1.37) |

Figure S2: Full phylogenies using (A) Nei’s *D* and (B) *δμ*^2^ based on microsatellite data, used for estimation of evolutionary rate for gill raker number.


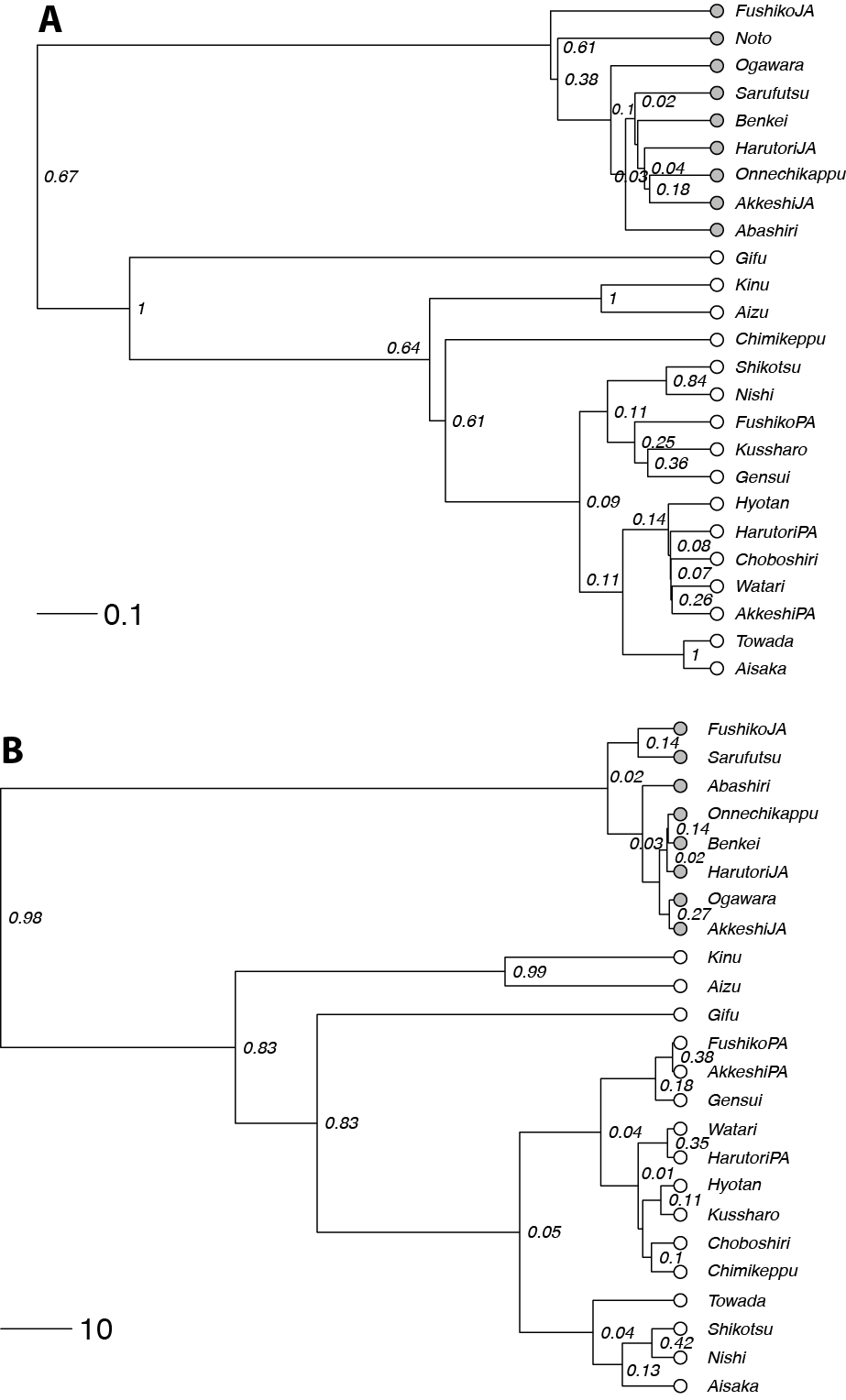


Figure S3: Log Likelihood ratio distributions for the single rate (red) and multiple rate (blue) Brownian motion models generated from 1000 simulated datasets using parametric bootstrapping. Dashed vertical line indicates position of log Likelihood ratio observed from the actual data.


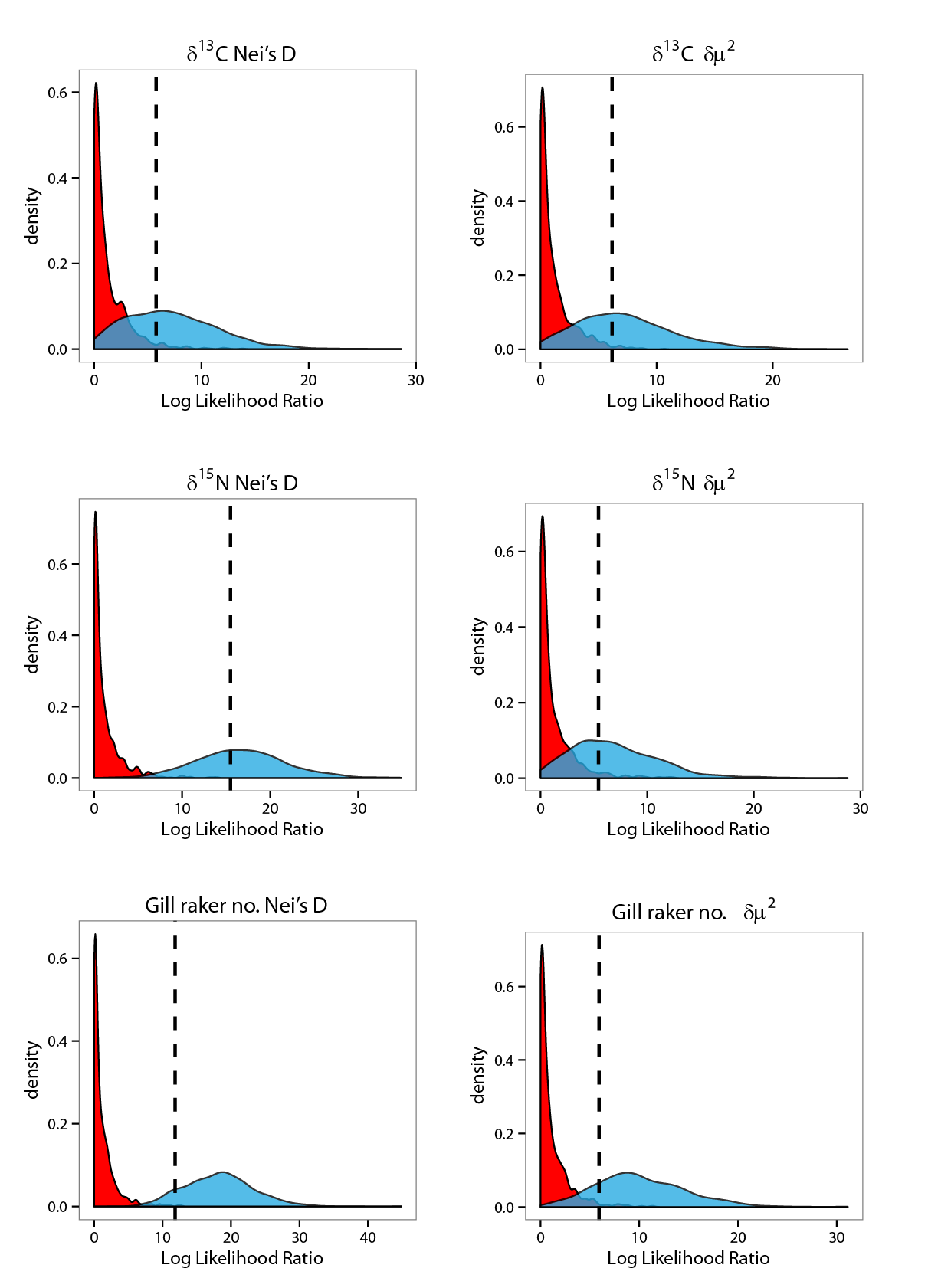


Figure S4: Log Likelihood ratio distributions for the single optima (red) and two optima (blue) Ornstein-Uhlenbeck models generated from 1000 simulated datasets using parametric bootstrapping. Dashed vertical line indicates position of log Likelihood ratio observed from the actual data.


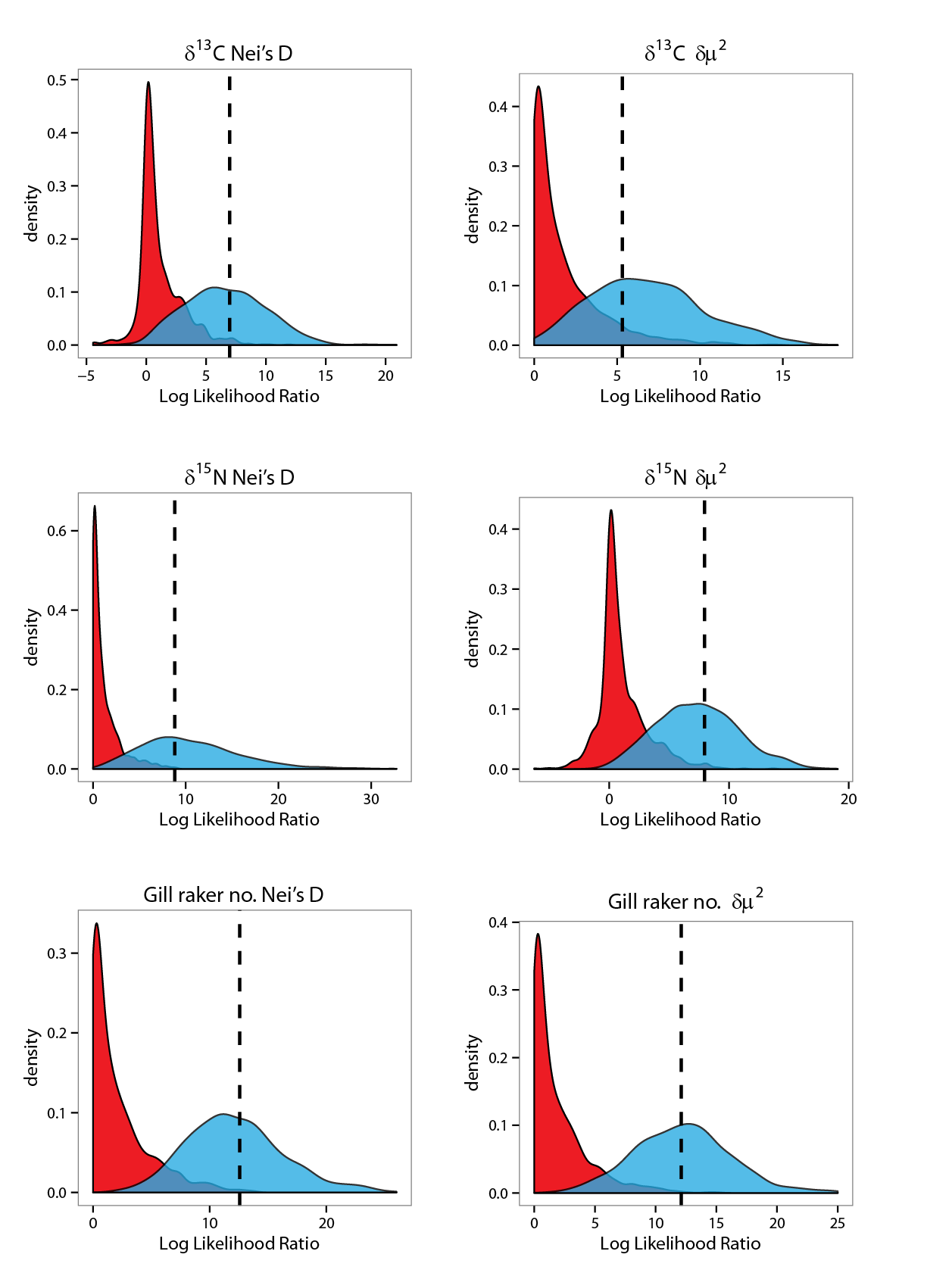


Figure S5: Log Likelihood ratio distributions for the two optima (red) and three optima (blue) Ornstein-Uhlenbeck models generated from 1000 simulated datasets using parametric bootstrapping. Dashed vertical line indicates position of log Likelihood ratio observed from the actual data.

**
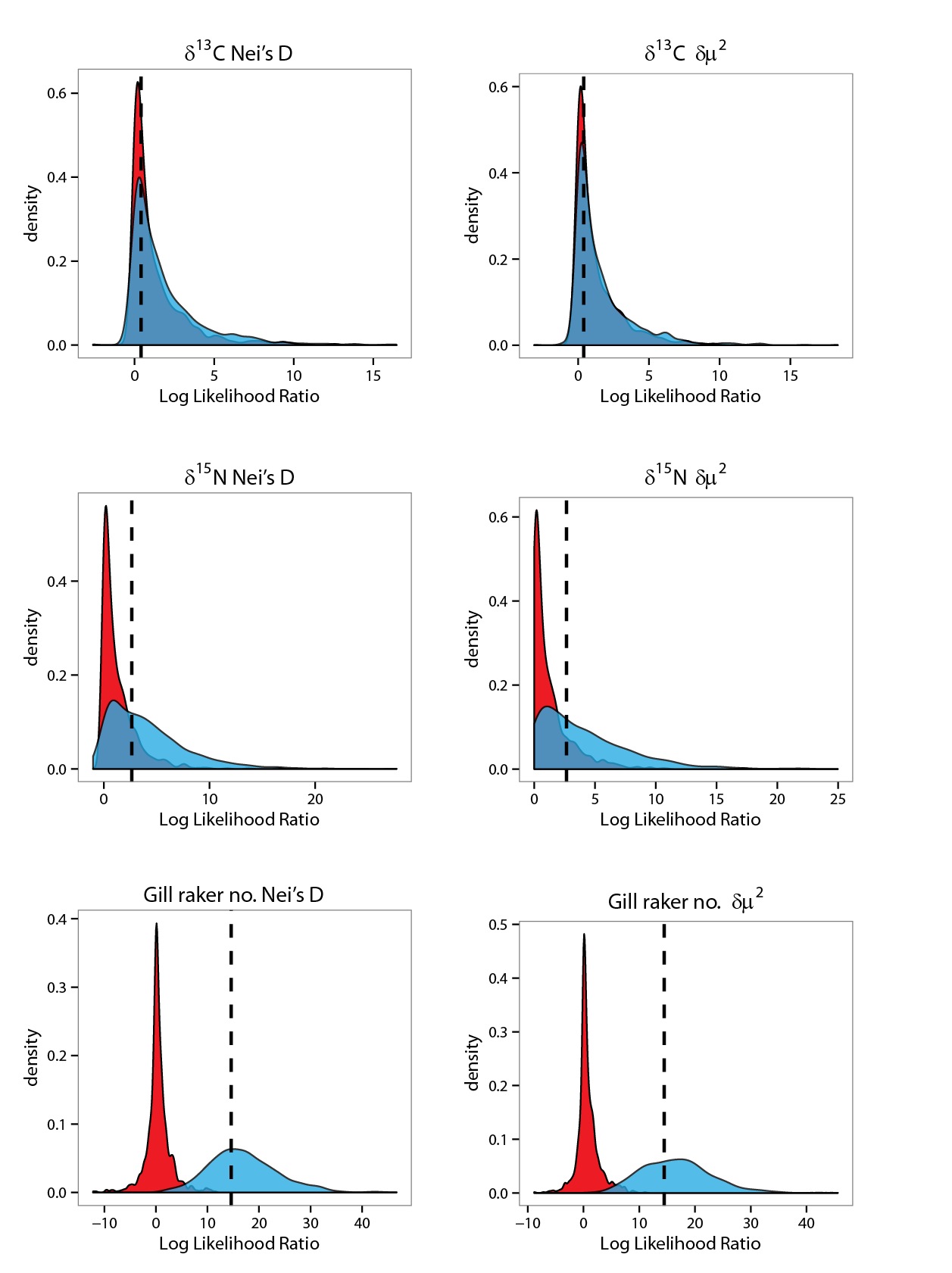
**

Figure S6: Log Likelihood ratio distributions for best supported Brownian motion (red) and Ornstein-Uhlenbeck (blue) models generated from 1000 simulated datasets using parametric bootstrapping. Dashed vertical line indicates position of log Likelihood ratio observed from the actual data. NB: All comparisons are multiple rate BM vs multiple rate OU with the exception of niche size, which is multiple rate BM vs single rate OU.


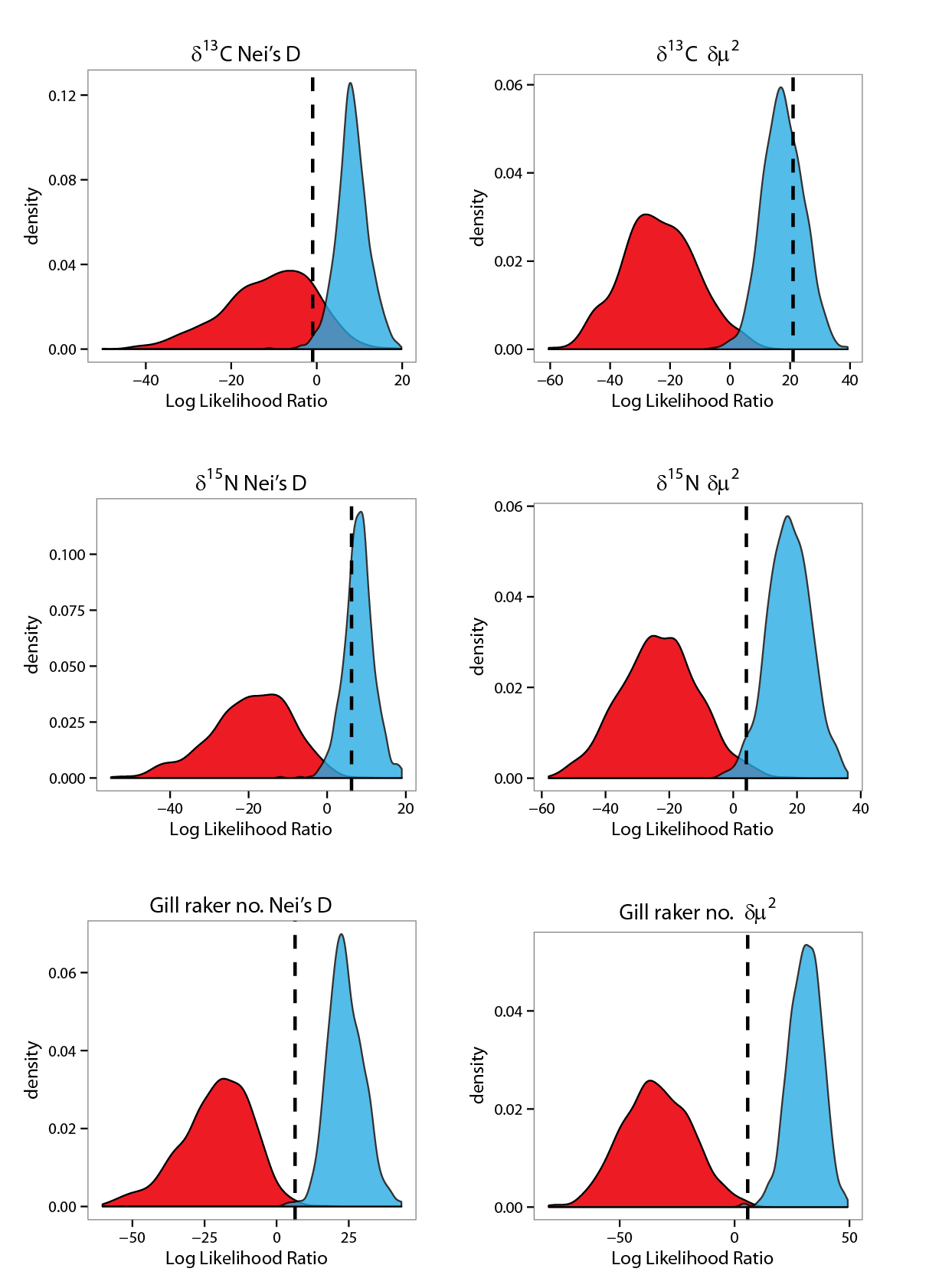


Table S7: Summary of power analysis based on parametric bootstrapping technique for Brownian motion and Ornstein-Uhlbeck models (see also S3 and S4). Observed LLR is observed Log Likelihood ratio between the single and multiple rate models, critical value is the critical Log Likelihood ratio required for the single rate model to be rejected at the α = 0.05 level, exact *P* is the exact *P*-value for rejection of the single rate model based on the simulated distributions and power is the power of the test to correctly reject the single rate model when the multiple model is true.

| Model | Trait | Phylogeny | Observed LLR | Critical value | Exact *P* | Power % |
| --- | --- | --- | --- | --- | --- | --- |
| BM1 v BM2 | δ^13^C | Nei’s *D* | 5.78 | 4.71 | 0.03 | 68.90 |
|  |  | *δμ*^2^ | 6.16 | 4.48 | 0.02 | 73.00 |
|  | δ^15^N | Nei’s *D* | 15.48 | 4.78 | 0.00 | 99.30 |
|  |  | *δμ*^2^ | 5.43 | 4.60 | 0.04 | 67.20 |
|  | Gill raker | Nei’s *D* | 11.84 | 4.71 | 0.00 | 99.90 |
|  |  | *δμ*^2^ | 5.93 | 4.41 | 0.02 | 90.70 |
| OU1 v OU2 | δ^13^C | Nei’s *D* | 6.98 | 4.73 | 0.02 | 69.70 |
|  |  | *δμ*^2^ | 5.31 | 6.37 | 0.07 | 52.30 |
|  | δ^15^N | Nei’s *D* | 8.81 | 4.79 | 0.00 | 86.90 |
|  |  | *δμ*^2^ | 7.96 | 5.02 | 0.01 | 74.50 |
|  | Gill raker | Nei’s *D* | 12.58 | 7.53 | 0.00 | 88.00 |
|  |  | *δμ*^2^ | 12.11 | 6.76 | 0.00 | 91.50 |
| OU2 v OU3 | δ^13^C | Nei’s *D* | 0.40 | 5.04 | 0.59 | 9.60 |
|  |  | *δμ*^2^ | 0.39 | 5.00 | 0.57 | 8.10 |
|  | δ^15^N | Nei’s *D* | 2.65 | 4.78 | 0.15 | 34.20 |
|  |  | *δμ*^2^ | 2.65 | 4.82 | 0.16 | 33.30 |
|  | Gill raker | Nei’s *D* | 14.59 | 4.24 | 0.00 | 98.30 |
|  |  | *δμ*^2^ | 14.47 | 4.62 | 0.00 | 99.20 |

Table S8: Summary of power analysis and model on BM v OU model comparisons; based on parametric bootstrapping technique for comparisons between Brownian motion and Ornstein-Uhlbeck models (see also S5). Observed LLR is observed Log Likelihood ratio between the single and multiple rate models, critical value is the critical Log Likelihood ratio required for the single rate model to be rejected at the α = 0.05 level, exact *P* is the exact *P*-value for rejection of the single rate model based on the simulated distributions and power is the power of the test to correctly reject the single rate model when the multiple model is true.

| Model | Trait | Phylogeny | Observed LLR | Critical value | Exact *P* | Power | Best |
| --- | --- | --- | --- | --- | --- | --- | --- |
| BM2 v OU2 | δ^13^C | Nei’s *D* | -0.93 | 4.29 | 0.99 | 88 | BM2 |
| BM2 v OU2 |  | *δμ*^2^ | 21.04 | -2.87 | 0.31 | 99.8 | OU2 |
| BM2 v OU2 | δ^15^N | Nei’s *D* | 6.22 | -4.02 | 0.72 | 99.7 | OU2 |
| BM2 v OU2 |  | *δμ*^2^ | 4.13 | -3.38 | 0.97 | 99.9 | OU2 |
| BM2 v OU3 | Gill raker | Nei’s *D* | 6.34 | -3.52 | 1 | 100 | - |
| BM2 v OU3 |  | *δμ*^2^ | 5.69 | -8.53 | 1 | 100 | - |

Table S9: Comparative estimates of rate of phenotypic evolution (σ^2^) under Ornstein-Uhlenbeck and Brownian motion models between Pacific Ocean (PO) and Japan Sea (JS) stickleback lineage for niche size and gill raker number; 95% CI = lower and upper 95% confidence intervals estimated using parametric bootstrapping with 1000 replicates.

| **OU1 model** | | | | | | | | | | | | | | | | | | | | | | | | | | |
| --- | --- | --- | --- | --- | --- | --- | --- | --- | --- | --- | --- | --- | --- | --- | --- | --- | --- | --- | --- | --- | --- | --- | --- | --- | --- | --- |
| Trait | | Phylogeny | | | α | | | 95% CI | | | | | σ^2^ | | | 95% CI | | | | | θ | | | 95% CI | | |
| δ^13^C | | Nei's D | | | 4.83 | | | 2.27-82.02 | | | | | 133.57 | | | 47.06-2307.82 | | | | | -20.15 | | | -22.50--18.02 | | |
|  | | δμ^2^ | | | 2.92 | | | 1.31-69.42 | | | | | 78.90 | | | 35.74-1682.29 | | | | | -20.03 | | | -22.63--17.46 | | |
| δ^15^N | | Nei's D | | | 74.63 | | | 50.54-213.80 | | | | | 655.57 | | | 651.89-569.35 | | | | | 11.54 | | | 10.65-12.47 | | |
|  | | δμ^2^ | | | 3.68 | | | 1.60-78.64 | | | | | 32.78 | | | 12.36-670.22 | | | | | 11.81 | | | 10.50-13.20 | | |
| Gill raker | | Nei's D | | | 2.29 | | | 1.18-82.52 | | | | | 45.98 | | | 24.62-995.21 | | | | | 22.12 | | | 19.34-24.76 | | |
|  | | δμ^2^ | | | 1.57 | | | 0.81-60.57 | | | | | 31.69 | | | 17.14-659.33 | | | | | 22.30 | | | 19.48-25.17 | | |
|  | | | | | | | | | | | | | | | | | | | | | | | | | | |
| **OU2 model** | | | | | | | | | | | | | | | | | | | | | | | | | | |
| Trait | | Phylogeny | | α | | | 95% CI | | | | σ^2^ | 95% CI | | | | | Θ_JS_ | | 95% CI | | | Θ_PO_ | | | 95% CI | |
| δ^13^C | | Nei's D | | 55.78 | | | 7.06-119.92 | | | | 1025.23 | 169.85-1873.78 | | | | | -17.70 | | -20.07--15.29 | | | -22.11 | | | -23.89--20.14 | |
|  | | δμ^2^ | | 10.26 | | | 3.49-78.78 | | | | 188.20 | 47.16-1556.23 | | | | | -17.69 | | -19.88--15.44 | | | -22.09 | | | -23.83--20.35 | |
| δ^15^N | | Nei's D | | 74.32 | | | 60.24-264.24 | | | | 410.57 | 411.23-608.97 | | | | | 13.21 | | 12.02-14.36 | | | 10.57 | | | 9.66-11.40 | |
|  | | δμ^2^ | | 68.15 | | | 55.05-238.60 | | | | 376.69 | 380.62-546.29 | | | | | 13.22 | | 11.97-14.44 | | | 10.57 | | | 9.64-11.54 | |
| Gill raker | | Nei's D | | 7.46 | | | 3.81-103.40 | | | | 66.29 | 23.24-920.54 | | | | | 24.2 | | 23.08-26.49 | | | 19.43 | | | 18.17-20.67 | |
|  | | δμ^2^ | | 4.75 | | | 2.40-69.49 | | | | 42.48 | 17.93-636.27 | | | | | 24.87 | | 23.07-26.85 | | | 19.46 | | | 18.18-20.67 | |
|  | | | | | | | | | | | | | | | | | | | | | | | | | | |
|  | | | | | | | | | | | | | | | | | | | | | | | | | | |
|  | | | | | | | | | | | | | | | | | | | | | | | | | | |
| **OU3 model** | | | | | | | | | | | | | | | | | | | | | | | | | | |
| Trait | Phylogeny | | α | | | 95% CI | | | σ^2^ | 95% CI | | | | Θ_JS_ | 95% CI | | | Θ_PO_ | | 95% CI | | | Θ_PF_ | | | 95% CI |
| δ^13^C | Nei's D | | 32.43 | | | 4.52-215.44 | | | 583.46 | 53.20-3973.11 | | | | -17.70 | -20.05--15.28 | | | -21.33 | | -24.33--18.34 | | | -22.53 | | | -27.33--19.85 |
|  | δμ^2^ | | 12.53 | | | 4.12-213.30 | | | 224.89 | 53.11-3897.35 | | | | -17.69 | -20.04--15.37 | | | -21.32 | | -24.25--18.34 | | | -22.80 | | | -26.47--19.83 |
| δ^15^N | Nei's D | | 212.80 | | | 151.12-726.20 | | | 1023.75 | 1025.74-1512.61 | | | | 13.22 | 12.13-14.35 | | | 11.64 | | 10.15-13.09 | | | 10.03 | | | 8.98-11.14 |
|  | δμ^2^ | | 94.42 | | | 6.97-362.69 | | | 54.09 | 35.95-994.96 | | | | 13.22 | 12.03-14.67 | | | 11.64 | | 10.21-13.20 | | | 10.03 | | | 8.25-11.12 |
| Gill raker | Nei's D | | 255.33 | | | 187.13-805.97 | | | 1217.144 | 1214.82-1725.99 | | | | 24.51 | 23.31-25.51 | | | 21.75 | | 20.51-22.95 | | | 18.04 | | | 17.03-18.94 |
|  | δμ^2^ | | 182.57 | | | 135.21-597.13 | | | 870.27 | 873.87-1244.54 | | | | 24.51 | 23.44-25.64 | | | 21.75 | | 20.50-22.99 | | | 18.04 | | | 17.07-19.05 |

| **BM models** |  |  | |  | | | |  |  |  |  |  |
| --- | --- | --- | --- | --- | --- | --- | --- | --- | --- | --- | --- | --- |
|  |  | Single rate | | Multiple rate | | | |  |  |  |  |  |
| Trait | Phylogeny | σ^2^ | 95% CI | σ^2^_PO_ | 95% CI | σ^2^_JS_ | 95% CI | Log(*L*)_SINGLE_ | Log(*L*)_MULTIPLE_ | Single AIC_C_ | Multiple AIC_C_ | P |
| δ^13^C | Nei's D | 17.11 | 7.19-27.13 | 25.44 | 7.76-47.79 | 3.81 | 0.65-8.35 | -50.45 | -47.56 | 105.65 | 102.73 | 0.02 |
|  | δμ^2^ | 2.75 | 1.23-4.39 | 4.03 | 1.39-7.79 | 0.57 | 0.10-1.29 | -61.6 | -58.52 | 127.96 | 124.65 | 0.01 |
| δ^15^N | Nei's D | 12.59 | 5.34-20.85 | 20.14 | 7.05-38.35 | 0.62 | 0.09-1.25 | -47.47 | -39.73 | 99.68 | 87.05 | <0.0001 |
|  | δμ^2^ | 0.32 | 0.14-0.55 | 0.48 | 0.17-0.90 | 0.08 | 0.01-0.17 | -41.4 | -38.68 | 87.55 | 84.97 | 0.02 |
| Gill raker no | Nei's D | 7.48 | 3.85-11.81 | 13.01 | 5.27-24.31 | 0.46 | 0.11-0.95 | -53.57 | -47.65 | 111.71 | 102.5 | <0.001 |
|  | δμ^2^ | 0.29 | 0.15-0.46 | 0.45 | 0.18-0.82 | 0.05 | 0.01-0.10 | -50.29 | -47.33 | 101.85 | 105.16 | 0.01 |

Figure S11: Ratio of Pacific Ocean and Japan Sea σ^2^ values for Brownian motion models with increasing number of Pacific Ocean populations included in the analysis. Ratio values greater than 1 indicate a higher σ^2^ and thus greater phenotypic diversification in the Pacific Ocean lineage compared to the Japan Sea.


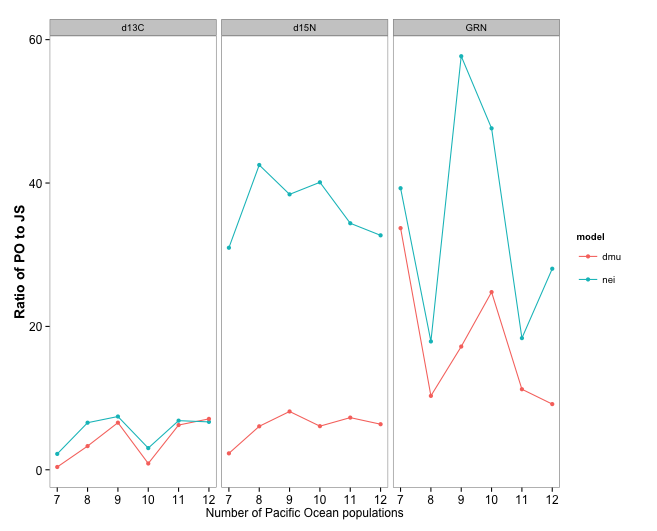


Figure S12: Ratio of Japan Sea and Pacific freshwater population θ values for Ornstein-Uhlbeck models with increasing number of Pacific Ocean populations included in the analysis. Ratio values greater than 1 indicate greater divergence in trait optima between the Japan Sea and Pacific Ocean freshwater populations.


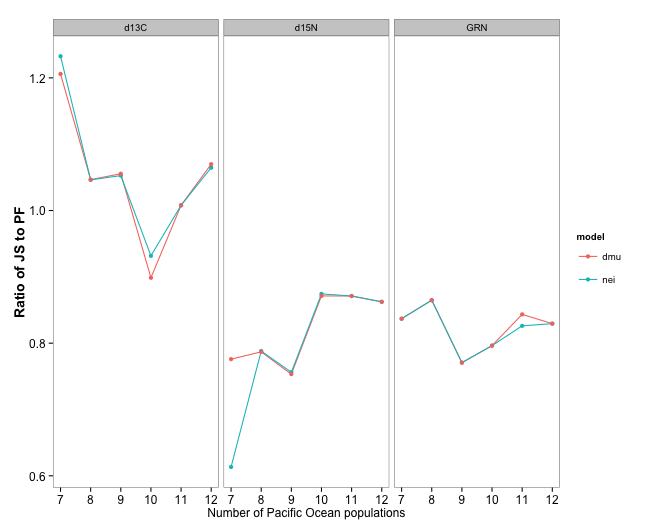


Figure S13: Curves indicating power to detect a difference between BM, OU and BM/OU model comparisons with increasing numbers of Pacific Ocean populations included in the analysis. Power estimates based on 100 parametric bootstrap comparisons.


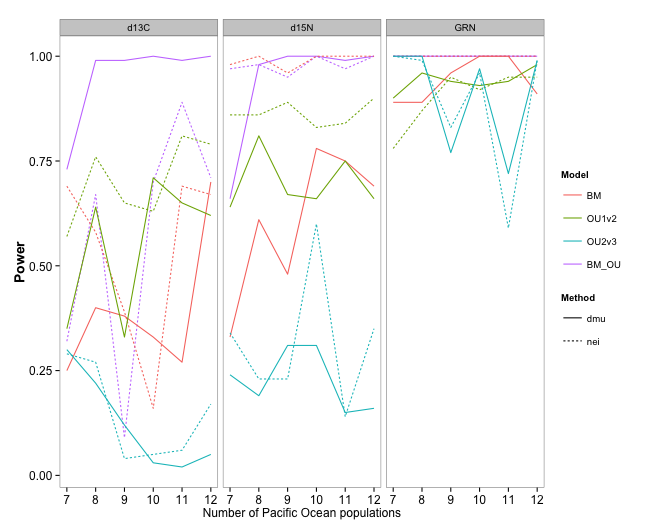


Text S14: Macroinvertebrate stable isotope variation

Marine stable isotope values did not vary between sites (δ^13^C: *P* = 0.66; δ^15^N: *P* = 0.44) and were pooled. Macroinvertebrate δ^13^C values became more depleted (see Table S9a) with increasing distance from the sea (GLMM, catchment and species as random factor, *R*^2^ = 0.96, *F*_3, 47_ = 6.10, *P* = 0.001), marking a transition from marine to freshwater carbon (Fig 4A). A similar pattern of clinal depletion was observed for mean δ^15^N values (*R*^2^ = 0.98, *F*_3, 47_ = 9.67, *P* < 0.0001, see Tables S14a and S14b).

Table S14a: Mean macroinvertebrate stable isotope data (grouped for Shiomi and Bekanbeushi river systems).

| Sample site | δ^13^C | SD | δ^15^N | SD |
| --- | --- | --- | --- | --- |
| Marine | -15.74 | 2.23 | 11.31 | 0.90 |
| Lower | -22.46 | 7.18 | 5.94 | 4.16 |
| Midstream | -26.88 | 3.53 | 8.61 | 2.58 |
| Upstream | -27.30 | 2.61 | 6.15 | 1.07 |

Table S14b: Summary table for GLMMs examining macroinvertebrate SIA variation within the Bekanbeushi and Shiomi river systems.

| Model | Fixed factor | Random factor | *R*^2^ | *F* | df | *P* |
| --- | --- | --- | --- | --- | --- | --- |
| δ^13^C |  |  |  |  |  |  |
| Bekanbeushi | Site | Species | 0.94 | 48.79 | 2, 28 | < 0.0001 |
| Shiomi | Site | Species | 0.96 | 6.66 | 3, 34 | 0.0001 |
| δ^15^N |  |  |  |  |  |  |
| Bekanbeushi | Site | Species | 0.97 | 30.12 | 3, 28 | < 0.0001 |
| Shiomi | Site | Species | 0.96 | 11.18 | 3, 34 | < 0.0001 |

Table S15: Dietary proportions for Japan Sea and Pacific Ocean fish in the Bekanbeushi system modelled using SIAR.

|  | Marine pelagic | | | | Marine benthic | | | | Freshwater benthic | | | |
| --- | --- | --- | --- | --- | --- | --- | --- | --- | --- | --- | --- | --- |
|  | Mean | Mode | L 95% HDR | U 95% HDR | Mean | Mode | L 95% HDR | U 95% HDR | Mean | Mode | L 95% HDR | U 95% HDR |
| Japan Sea | 0.49 | 0.45 | 0.33 | 0.66 | 0.40 | 0.43 | 0.28 | 0.53 | 0.11 | 0.11 | 0.06 | 0.16 |
| Pacific Ocean | 0.49 | 0.48 | 0.34 | 0.65 | 0.13 | 0.13 | 0.01 | 0.24 | 0.38 | 0.38 | 0.34 | 0.42 |
| JA bay | 0.44 | 0.47 | 0.24 | 0.75 | 0.43 | 0.40 | 0.20 | 0.58 | 0.13 | 0.13 | 0.04 | 0.20 |
| PA bay | 0.56 | 0.56 | 0.40 | 0.72 | 0.40 | 0.39 | 0.27 | 0.52 | 0.04 | 0.05 | 0.00 | 0.09 |
| JA lake | 0.38 | 0.42 | 0.20 | 0.69 | 0.35 | 0.32 | 0.11 | 0.49 | 0.27 | 0.26 | 0.19 | 0.33 |
| JA mid | 0.49 | 0.50 | 0.26 | 0.75 | 0.22 | 0.20 | 0.00 | 0.37 | 0.31 | 0.31 | 0.23 | 0.39 |
| PA mid | 0.50 | 0.47 | 0.27 | 0.66 | 0.11 | 0.13 | 0.00 | 0.28 | 0.39 | 0.40 | 0.33 | 0.46 |
| PA upstream | 0.45 | 0.43 | 0.23 | 0.61 | 0.08 | 0.12 | 0.00 | 0.28 | 0.45 | 0.44 | 0.38 | 0.51 |

JA, Japan Sea anadromous; PA, Pacific Ocean anadromous; L 95% HDR, lower 95% highest density region; U 95% HDR, upper 95% highest density region.

Figure S16: SIAR modelled contributions to diet in the Bekanbeushi system; boxes represent 95%, 75% and 25% posterior probability densities.


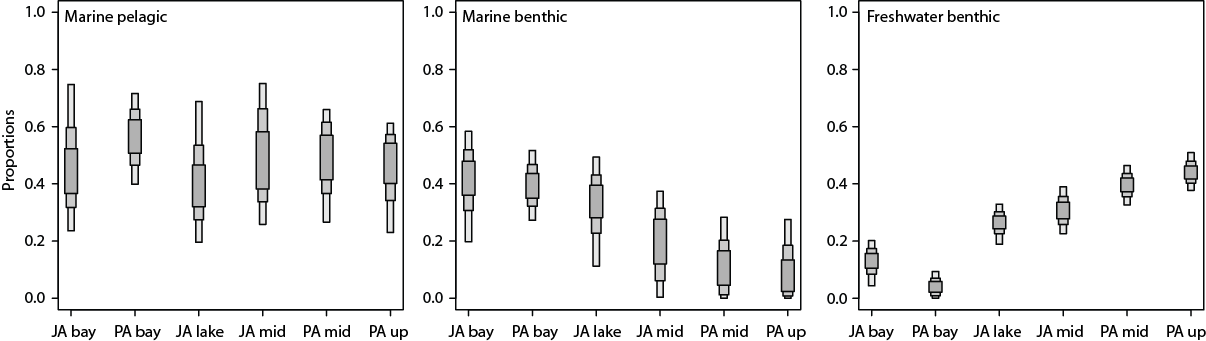


Table S17: Pairwise tests of differences between modelled SIAR outputs. Lower and upper diagonals represent alternative tails of same test (i.e. proportion of modelled output for population x greater than or less than modelled output for population y); emboldened text represents significance after correction for False Discovery Rate (B-Y method), † represents significance after Bonferroni correction; α = 0.05, α_Bonferroni_ = 0.003, α_FDR_ = 0.015. JA, Japan Sea anadromous; PA, Pacific Ocean anadromous.

| Marine pelagic | JA bay | PA bay | JA lake | JA mid | PA mid | PA upstream |
| --- | --- | --- | --- | --- | --- | --- |
| JA bay | - | 0.270 | 0.618 | 0.441 | 0.486 | 0.571 |
| PA bay | 0.730 | - | 0.824 | 0.644 | 0.735 | 0.828 |
| JA lake | 0.382 | 0.176 | - | 0.336 | 0.364 | 0.445 |
| JA mid | 0.559 | 0.356 | 0.664 | - | 0.558 | 0.637 |
| PA mid | 0.514 | 0.265 | 0.636 | 0.442 | - | 0.593 |
| PA upstream | 0.429 | 0.172 | 0.555 | 0.364 | 0.407 | - |
| Marine benthic | JA bay | PA bay | JA lake | JA mid | PA mid | PA upstream |
| JA bay | - | 0.546 | 0.748 | 0.920 | 0.973 | 0.976 |
| PA bay | 0.454 | - | 0.754 | 0.946 | 0.991 | 0.991 |
| JA lake | 0.252 | 0.246 | - | 0.803 | 0.914 | 0.927 |
| JA mid | 0.080 | 0.054 | 0.197 | - | 0.675 | 0.701 |
| PA mid | 0.027 | **0.009**† | 0.086 | 0.325 | - | 0.538 |
| PA upstream | 0.024 | **0.009**† | 0.073 | 0.299 | 0.462 | - |
| Freshwater benthic | JA bay | PA bay | JA lake | JA mid | PA mid | PA upstream |
| JA bay | - | 0.947 | **0.006**† | **0.000**† | **0.000**† | **0.000**† |
| PA bay | 0.053 | - | **0.000**† | **0.000**† | **0.000**† | **0.000**† |
| JA lake | 0.994 | 1.000 | - | 0.204 | **0.002**† | **0.000**† |
| JA mid | 1.000 | 1.000 | 0.796 | - | 0.055 | **0.007**† |
| PA mid | 1.000 | 1.000 | 0.998 | 0.945 | - | 0.169 |
| PA upstream | 1.000 | 1.000 | 1.000 | 0.993 | 0.831 | - |

Table S18: Bayesian isotopic niche-width metrics estimated from δ^13^C and δ^15^N values for Bekanbeushi Japan Sea and Pacific Ocean fish, modelled using SIBER; SEA^­^_C_ = corrected Standard Ellipse Area, SEA^­^_B_ = Bayesian Standard Ellipse Area. JA, Japan Sea anadromous; PA, Pacific Ocean anadromous.

|  | SEA_C_ | SEA_B_ (±SD) |
| --- | --- | --- |
| Japan Sea | 1.31 | 1.49 (0.22) |
| Pacific Ocean | 0.75 | 0.92 (0.12) |
| JA bay | 0.93 | 1.72 (0.44) |
| PA bay | 0.22 | 0.56 (0.13) |
| JA lake | 0.50 | 0.89 (0.20) |
| JA midstream | 1.58 | 2.24 (0.75) |
| PA midstream | 0.34 | 0.71 (0.17) |
| PA upstream | 0.74 | 1.09 (0.25) |

Table S19: Pairwise niche overlap estimates based on SEA_C_ values for Bekanbeushi Pacific Ocean and Japan Sea stickleback. JA, Japan Sea anadromous; PA, Pacific Ocean anadromous.

|  | JA bay | PA bay | JA lake | JA midstream | PA midstream | PA upstream |
| --- | --- | --- | --- | --- | --- | --- |
| JA bay | - |  |  |  |  |  |
| PA bay | 0.00 | - |  |  |  |  |
| JA lake | 0.23 | 0.00 | - |  |  |  |
| JA midstream | 0.50 | 0.00 | 0.02 | - |  |  |
| PA midstream | 0.00 | 0.00 | 0.00 | 0.00 | - |  |
| PA upstream | 0.00 | 0.00 | 0.00 | 0.00 | 0.22 | - |

Table S20: Dietary composition and indices of relative importance for Japan Sea and Pacific Ocean stickleback and for forms at different sites within the Bekanbeushi system. %F = frequency of occurrence, %N = percentage abundance based on numbers of prey items, %W = percentage abundance based on weight of prey items, %I = index of relative importance (overleaf).

|  | JA | | | | PA | | | | JA | | | | JA | | | | PA - midstream | | | | PA | | | |
| --- | --- | --- | --- | --- | --- | --- | --- | --- | --- | --- | --- | --- | --- | --- | --- | --- | --- | --- | --- | --- | --- | --- | --- | --- |
| Prey items | (n = 106) | | | | (n = 178) | | | | lake  (n = 97) | | | | midstream  (n = 9) | | | | midstream  (n = 111) | | | | upstream  (n = 67) | | | |
|  | %F | %N | %W | %I | %F | %N | %W | %I | %F | %N | %W | %I | %F | %N | %W | %I | %F | %N | %W | %I | %F | %N | %W | %I |
| Terrestrial | 1.51 | 0.39 | 0.79 | 1.22 | 0.56 | 0.02 | 0.18 | 0.08 | 1.65 | 0.41 | 0.90 | 1.40 | 0.00 | 0.00 | 0.00 | 0.00 | 0.00 | 0.00 | 0.00 | 0.00 | 1.49 | 0.09 | 0.39 | 0.58 |
| Zooplankton | 9.25 | 6.69 | 4.91 | 73.35 | 0.79 | 0.23 | 0.03 | 0.14 | 9.59 | 6.85 | 5.53 | 77.29 | 5.56 | 3.61 | 0.23 | 16.91 | 0.99 | 0.29 | 0.05 | 0.21 | 0.45 | 0.01 | 0.01 | 0.01 |
| Benthos | 5.47 | 0.52 | 1.74 | 8.45 | 9.33 | 7.21 | 6.09 | 83.70 | 5.26 | 0.34 | 0.97 | 4.49 | 7.78 | 3.84 | 7.49 | 69.86 | 9.46 | 8.43 | 6.86 | 89.75 | 9.10 | 3.07 | 5.21 | 60.76 |
| Fish | 0.85 | 0.08 | 0.25 | 0.19 | 0.06 | 0.00 | 0.06 | 0.00 | 0.93 | 0.08 | 0.28 | 0.22 | 0.00 | 0.00 | 0.00 | 0.00 | 0.00 | 0.00 | 0.00 | 0.00 | 0.15 | 0.01 | 0.13 | 0.02 |
| Fish eggs | 4.53 | 1.03 | 1.57 | 8.04 | 3.88 | 2.39 | 1.64 | 10.55 | 4.64 | 0.96 | 1.60 | 7.72 | 3.33 | 2.38 | 1.32 | 9.79 | 3.60 | 1.17 | 1.20 | 5.31 | 4.33 | 6.55 | 2.14 | 30.32 |
| Plant material | 1.60 | 0.02 | 0.15 | 0.19 | 4.04 | 0.08 | 0.66 | 2.03 | 1.44 | 0.02 | 0.11 | 0.12 | 3.33 | 0.07 | 0.48 | 1.45 | 3.24 | 0.05 | 0.37 | 0.85 | 5.37 | 0.17 | 1.00 | 5.07 |
| Other | 6.70 | 1.27 | 0.60 | 8.56 | 3.71 | 0.07 | 1.33 | 3.51 | 6.91 | 1.33 | 0.62 | 8.76 | 4.44 | 0.09 | 0.47 | 1.99 | 3.96 | 0.06 | 1.52 | 3.88 | 3.28 | 0.10 | 1.12 | 3.24 |

Table S21: Mean (± SD) foraging efficiency metrics for Japan Sea and Pacific Ocean anadromous stickleback.

|  | Japan Sea | | Pacific Ocean | |
| --- | --- | --- | --- | --- |
|  | Mean | SD | Mean | SD |
| Standard length (mm) | 53.59 | 2.40 | 68.24 | 3.47 |
| Latency (secs) | 97.13 | 48.35 | 133.05 | 137.33 |
| Number of vertical strikes | 29.40 | 17.83 | 24.84 | 12.61 |
| Number of horizontal strikes | 26.47 | 27.99 | 19.37 | 13.31 |
| Number of prey items handled | 14.33 | 7.93 | 14.26 | 8.07 |
| Number of prey items consumed | 3.00 | 2.54 | 8.37 | 4.10 |
| Efficacy | 0.08 | 0.03 | 0.27 | 0.17 |
| Strikes sec^-1^ | 0.05 | 0.03 | 0.04 | 0.02 |

Table S22: Mean gill raker number, sample sizes (*n*) and locations for all populations, Pacific Ocean anadromous (PA), Japan Sea anadromous (JA) and Pacific Ocean freshwater (PF).

| Population | Form | Gill raker no. | SD | *n* | Latitude | Longitude |
| --- | --- | --- | --- | --- | --- | --- |
| Abashiri | JA | 24.95 | 1.93 | 20 | 43.960 | 144.200 |
| Aisaka | PF | 17.00 | 1.73 | 7 | 40.592 | 141.221 |
| Akkeshi JA | JA | 24.98 | 1.3 | 44 | 43.068 | 144.884 |
| Akkeshi PA | PA | 21.46 | 1.33 | 28 | 43.105 | 144.891 |
| Fushiko JA | JA | 23.00 | 1.55 | 11 | 42.540 | 141.333 |
| Fushiko PA | PA | 21.29 | 1.44 | 14 | 42.540 | 141.333 |
| Harutori JA | JA | 25.35 | 1.76 | 20 | 42.969 | 144.396 |
| Kussharo | PF | 19.05 | 4.37 | 20 | 43.600 | 144.348 |
| Ogawara | JA | 24.46 | 1.76 | 13 | 40.840 | 141.372 |
| Onnechikappu | JA | 24.55 | 1.28 | 20 | 42.966 | 144.116 |
| Sarufutsu | JA | 24.25 | 1.71 | 20 | 45.255 | 142.239 |
| Shikotsu | PF | 15.40 | 1.98 | 20 | 42.775 | 141.400 |
| Towada | PF | 19.18 | 1.31 | 38 | 40.445 | 140.842 |
| Aizu | PF | 16.23 | 0.84 | 31 | 37.511 | 139.866 |
| Chimikeppu | PF | 20.80 | 1.4 | 20 | 43.629 | 143.885 |
| Gensui | PF | 19.75 | 1.41 | 20 | 39.365 | 141.897 |
| Kinugawa | PA | 17.90 | 1.32 | 13 | 37.511 | 139.866 |
| Nishitappu | PF | 21.28 | 1.2 | 40 | 42.646 | 141.476 |
| Choboshi | PA | 22.50 | 1.73 | 20 | 43.258 | 145.556 |
| Harutori PO | PA | 21.62 | 1.1 | 33 | 42.969 | 144.396 |
| Hyotan | PA | 21.52 | 1.04 | 17 | 43.032 | 144.844 |
| Watarichippu | PA | 22.10 | 1.37 | 20 | 43.036 | 145.053 |
| Benkei | JA | 24.50 | 1.73 | 12 | 42.825 | 140.188 |
